# Epigenetic reprogramming of fallopian tube fimbriae in *BRCA* mutation carriers defines early ovarian cancer evolution

**DOI:** 10.1038/ncomms11620

**Published:** 2016-05-24

**Authors:** Thomas E. Bartlett, Kantaraja Chindera, Jacqueline McDermott, Charles E. Breeze, William R. Cooke, Allison Jones, Daniel Reisel, Smita T. Karegodar, Rupali Arora, Stephan Beck, Usha Menon, Louis Dubeau, Martin Widschwendter

**Affiliations:** 1 Department of Women's Cancer, UCL Elizabeth Garrett Anderson Institute for Women's Health, University College London, London WC1E 6AU, UK; 2 Department of Statistical Science, University College London, London WC1E 7HB, UK; 3 Department of Medical Genomics, UCL Cancer Institute, University College London, London WC1E 6BT, UK; 4 Department of Pathology, UCL Cancer Institute, University College London, London WC1E 6BT UK; 5 Department of Pathology, USC/Norris Comprehensive Cancer Center, Keck School of Medicine of University of Southern California, Los Angeles, California 90033, USA

## Abstract

The exact timing and contribution of epigenetic reprogramming to carcinogenesis are unclear. Women harbouring *BRCA1/2* mutations demonstrate a 30–40-fold increased risk of high-grade serous extra-uterine Müllerian cancers (HGSEMC), otherwise referred to as ‘ovarian carcinomas', which frequently develop from fimbrial cells but not from the proximal portion of the fallopian tube. Here we compare the DNA methylome of the fimbrial and proximal ends of the fallopian tube in *BRCA1/2* mutation carriers and non-carriers. We show that the number of CpGs displaying significant differences in methylation levels between fimbrial and proximal fallopian tube segments are threefold higher in *BRCA* mutation carriers than in controls, correlating with overexpression of activation-induced deaminase in their fimbrial epithelium. The differentially methylated CpGs accurately discriminate HGSEMCs from non-serous subtypes. Epigenetic reprogramming is an early pre-malignant event integral to *BRCA1/2* mutation-driven carcinogenesis. Our findings may provide a basis for cancer-preventative strategies.

## Abstract (web summary)

Women with germline variants in BRCA genes are predisposed to ovarian cancer. In this study, the authors demonstrate that fimbrial tissue from the ovary, the site of ovarian cancer, in *BRCA* mutant carriers contains marked DNA methylation changes compared with the proximal region of the ovary.

## Graphical abstract

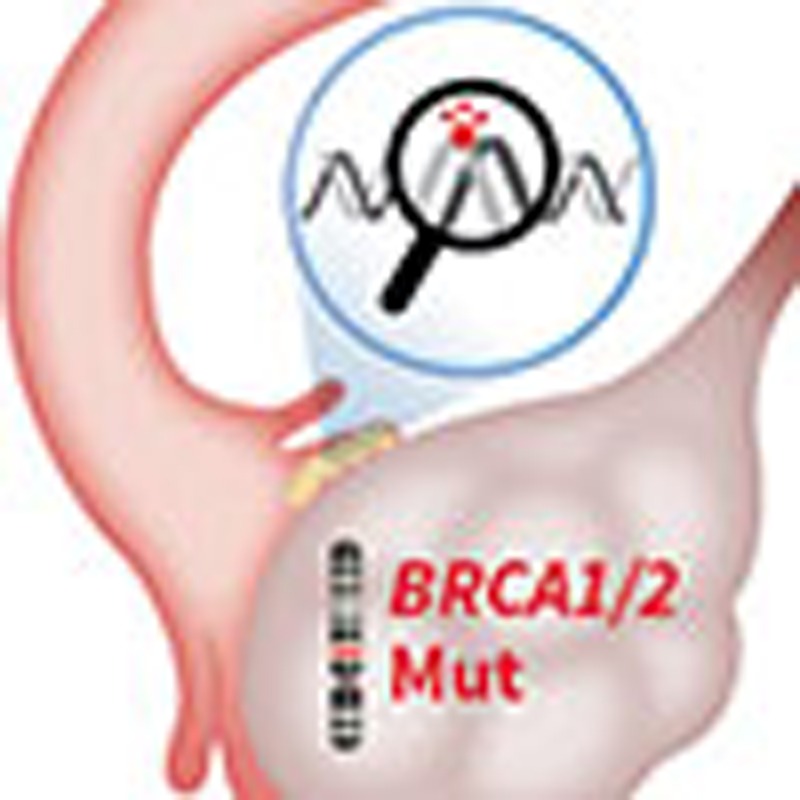

The development of non-invasive primary cancer preventive measures in humans requires a thorough understanding of the initial cancer-driving molecular mechanisms. High-grade serous extra-uterine Müllerian cancers (HGSEMC) (formerly classified as ovarian/tubal/peritoneal) present at late stage and are associated with <40% 5-year survival. Although the recent TCGA (The Cancer Genome Atlas) initiatives^1^ revealed key molecular changes in established cancers, very little is known about the initial molecular alterations in HGSEMC development. Analyses of tissues carrying an increased risk before the development of any microscopic alteration are lacking. *BRCA1/2* mutation carriers have up to a 30–40-fold increased risk of developing reproductive cancers^2^,^3^,^4^,^5^, predominantly HGSEMC^6^ and breast cancer. Despite a plethora of evidence linking mutations in *BRCA1* or *BRCA2* to cancer development^7^ the core components, such as organ specificity (that is, to breast and fallopian tube), are still poorly understood. The fallopian tube of *BRCA* mutation carriers offers a unique opportunity to study HGSEMC development because these cancers originate only from the distal (that is, fimbrial) end of the fallopian tube (close to the ovary), and not from the proximal end (close to the uterus)^8^,^9^,^10^. The ovary, being in close proximity to the fimbriae, is usually infiltrated early by invasive fimbrial cancers, accounting for their frequent classification as ovarian carcinomas.

Current evidence supporting the notion that epigenetic reprogramming is a crucial step in cancer development^11^ is largely based on artificial model systems^12^ or on association studies in established cancers^13^. Specifically, enhancer and repressed regions marked in each cell type with histone 3 lysine 4 monomethylation (H3K4me1) and histone 3 lysine 27 trimethylation (H3K27me3), respectively, are subject to reprogramming at the level of histone marks and the DNA methylome^11^,^14^.

Here we describe an epigenetic reprogramming phenomenon occurring specifically in the proximal end of the fallopian tube in *BRCA* mutation carriers, which is not evident in matched controls. Furthermore, we show that this epigenetic reprogramming event is driven by aberrantly high AID (activation-induced cytosine deaminase) expression and is an integral early pre-malignant event in HGSEMC development. Our findings provide the basis for the development of primary ovarian cancer prevention strategies in *BRCA1/2* mutation carriers.

## Results

### DNA methylation in fimbrial and proximal fallopian tube

**Figure 1:**
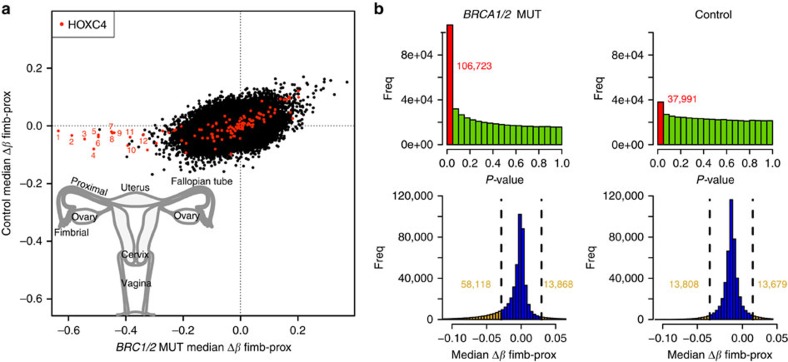
Differences in methylation profiles of the proximal versus fimbrial fallopian tube compartments in *BRCA* mutation carriers and controls. (**a**) Scatter-plot showing methylation differences (Δ*β*) between the fimbrial and proximal (fimb-prox) compartments of fallopian tubes in control volunteers (*n*=49) and *BRCA1*/*2* mutation carriers (*n*=52). The 12 CpGs located at the *HOXC4* locus and showing the largest methylation difference in *BRCA1*/*2* mutation carriers are indicated in red and numbered. (**b**) (top) *T*-test *p*-value histograms showing concentrations of significant CpGs discriminating fimbrial from proximal compartments (one *p*-value per CpG); (bottom) histograms of median Δ*β* fimb-prox (across all samples, one median per CpG) showing the magnitude of *β* differences between fimbrial and proximal compartments.

We performed epigenome-wide DNA methylation (DNAme) analyses (using the Illumina 450k DNA methylation bead-array assay assessing DNAme at ∼480,000 CpG sites) in 215 microscopic foci of normal fallopian tubes from *BRCA1/2* mutation carriers (*n*=56) and controls (*n*=59) who had their tubes/ovaries removed for risk-reduction or other reasons, respectively. We analysed both the fimbrial (at elevated risk) and the proximal (at average risk) portions of the tubes separately (Fig. 1a) in 52 carriers and 49 controls in order to adjust for epigenetic effects of no immediate importance to the carcinogenic process. Whereas only 37,991 CpGs demonstrated significant differences between the fimbrial and proximal segments in controls, 106,723 CpGs demonstrated significant differences between these two sites in *BRCA1/2* mutation carriers (Fig. 1b). Of those CpGs demonstrating >0.03 median *β* value difference between the fimbrial compared with the proximal segments, 81 and 19% were hypo- and hypermethylated, respectively (Fig. 1a,b and Supplementary Data 1 and 2). These figures remained unchanged irrespective of whether the mutation was in *BRCA1* or *BRCA2* (Supplementary Figs 1 and 2).

### Specific histone modification marks in reprogrammed regions

**Figure 2:**
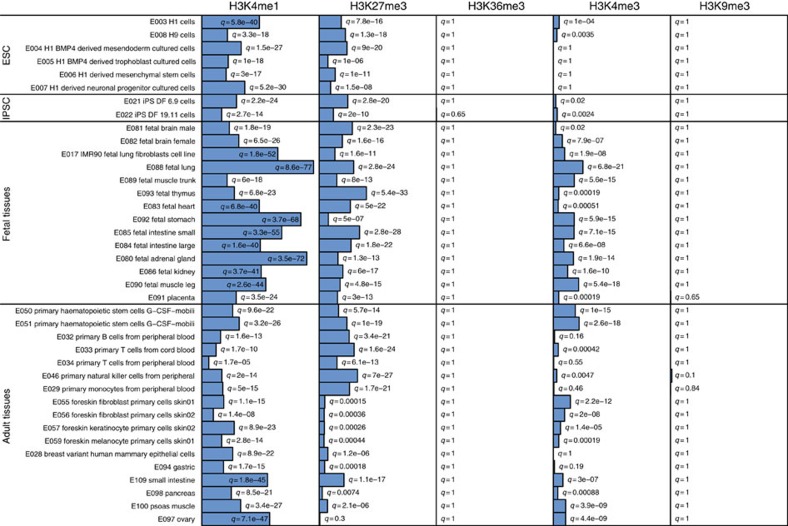
Enrichment of largest fimbrial versus proximal differences by major histone modification sites. eFORGE *q*-values for overlap between the 1,000 CpGs showing the greatest hypomethylation differences between fimbrial and proximal compartments in *BRCA* mutation carriers (but not in controls) and H3K4me1, H3K27me3, H3K36me3, H3K4me3 and H3K9me3 peaks from the Roadmap Epigenomics consortium (consolidated epigenomes release 2015, covering 39 tissues). For each set of test DMPs (differentially methylated positions), an overlap analysis is performed against the functional elements from either data source for each cell sample separately (ENCODE and Roadmap), and the number of overlaps is counted. A background distribution of the expected overlap counts for this DMP set is obtained by picking sets of the same number of DMPs as the test DMP set, matched for gene relationship and CpG island relationship annotation. The matched background sets are then overlapped with the functional elements and the background distribution of overlaps determined. By default 1,000 matched sets are used. The enrichment value for the test DMP set is plotted as the −log10 (binomial *p*-value). The Benjamini–Yekutieli (BY) multiple-testing corrected *q*-value is evaluated to mark enrichments as significant at *q*<0.05.

As expected^15^,^16^,^17^, gene set enrichment analysis (GSEA) demonstrates that those CpGs that are hypermethylated in the fimbrial versus proximal end of the fallopian tube in *BRCA1/2* mutation carriers are associated with cancer development (Supplementary Data 3) and are notably enriched for cancer or stem cell genes and genes targeted by EED (a component of the polycomb repressor complex 2, PRC2) or marked by H3K27me3. Unexpectedly, genes that are significantly hypomethylated in the fimbrial versus proximal segments in mutation carriers (but not in controls) are also enriched for PRC2 targets, including H3K27me3-marked sites (Supplementary Data 4). We applied the eFORGE tool (http://eforge.cs.ucl.ac.uk/) to explore whether there are specific regions prone to preferential epigenetic reprogramming within the fimbrial tissue of *BRCA1/2* carriers to document tissue-specific patterns of histone modification. Hypomethylated regions were shown to be enriched for highly tissue-specific H3K4me1 marks, whereas regions containing the repressive H3K27me3 mark were enriched preferentially for undifferentiated and fetal states as indicated by the particularly low average *q*-values (that is, *p*-values corrected for false discovery rate (FDR)) in stem cells and fetal tissues (Fig. 2). A similar pattern was observed for hypermethylated regions (Supplementary Fig. 3).

### HOXC4 methylation and expression

**Figure 3:**
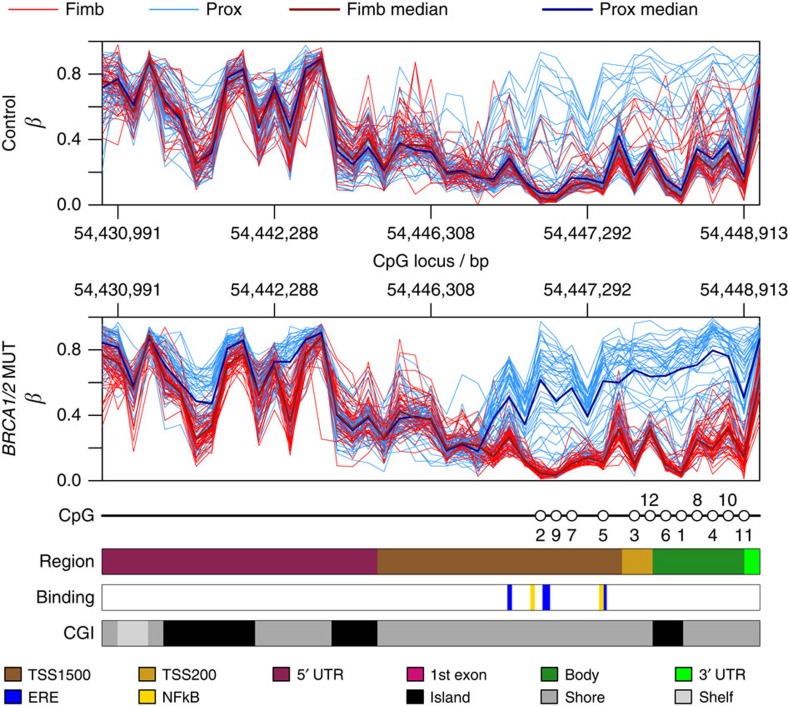
Methylation profile across *HOXC4* for control and *BRCA1*/*2* mutant samples. Control (*n*=49), *BRCA1/2* (*n*=52). CpGs numbered in Fig. 1a are again indicated here. Genomic regions are shown, together with CpG island information and NFkB- and ERE- (estrogen response element) binding sites.

**Figure 4:**
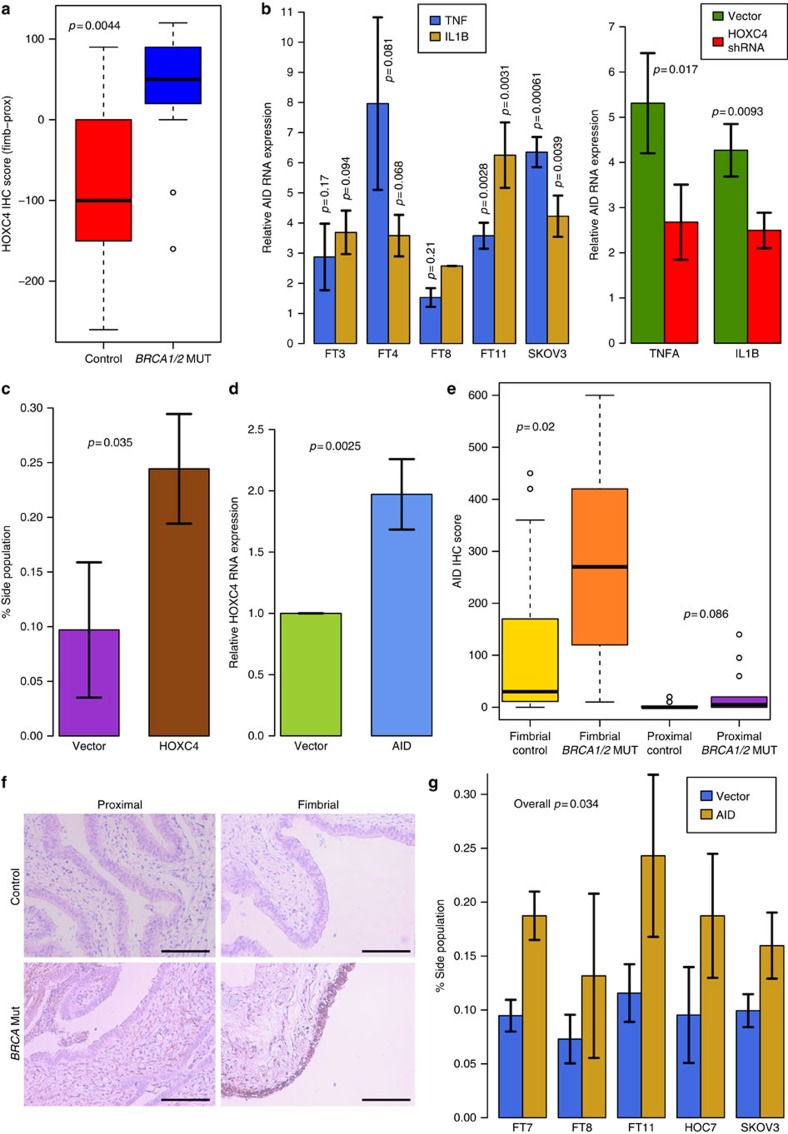
Functional relevance of HOXC4 and AID in the fallopian tube. (**a**) FFPE sections of proximal and fimbrial compartments of fallopian tubes from *BRCA* carriers (*n*=13) and non-carriers (*n*=14) were stained with an anti-HOXC4 antibody and scored for signal intensity. *P*-values were calculated with the Wilcox-test; the box shows the upper and lower quartiles and the whiskers show 1.5 X inter-quartile range beyond the upper and lower quartiles. (**b**, left) Primary cultures of secretory epithelial cells isolated from the fimbrial compartment of the fallopian tube (FT3, FT4, FT8 and FT11) and long-term cultures of SKOV3 ovarian carcinoma cells were treated with TNF-α or IL1-β for 6 h. Relative expression of AID mRNA was analysed by real-time PCR; data from three replicates are shown. (right) Relative expression of AID mRNA determined by real-time PCR in SKOV3 cells stably expressing HOXC4 shRNA or transfected with an empty vector and treated with TNF-α or IL1-β for 6 h; data from three replicates are shown. *P*-values were calculated with the *t*-test; the error bars indicate one standard deviation. (**c**) SKOV3 cells stably overexpressing HOXC4 or empty vector were treated with Hoechst 3342 (5 μg ml^−1^) for 90 min and Hoechst low-staining cells (side population) were examined by flow cytometry; data from three replicates are shown. *P*-values were calculated with the *t*-test; the error bars indicate 1 s.d. (**d**) HOXC4 mRNA expression determined by real-time PCR in SKOV3 cells stably overexpressing AID or transfected with an empty vector; data from three replicates are shown. *P*-values were calculated with the *t*-test; the error bars indicate 1 s.d. (**e**) AID protein expression in FFPE sections of proximal and fimbrial compartments of the fallopian tube of BRCA carriers (*n*=13) and controls (*n*=14) analysed by immunohistochemistry and scored for signal intensity. *P*-values were calculated with the Wilcox-test; the boxes show the upper and lower quartiles and the whiskers show 1.5 X inter-quartile range beyond the upper and lower quartiles. (**f**) Representative AID immunostains (× 200 magnification). Bars indicate 200 μm. (**g**) Primary cultures of secretory epithelial cells from the fimbrial fallopian tube compartment (FT7, FT8 and FT11), and of immortal cultures of HOC7 and SKOV3 ovarian carcinoma cells stably overexpressing AID or transfected with an empty vector were treated with Hoechst 3342 (5 μg ml^−1^) for 90 min and analysed by flow cytometry to evaluate the Hoechst low-staining cells (side population); data from three replicates are shown. *P*-values were calculated with the *t*-test; the error bars indicate 1 s.d.

CpGs showing the largest methylation difference between fimbrial and proximal compartments in mutation carriers belonged to the *HOXC4* gene (Fig. 1a). Whereas the majority of the 44 *HOXC4* CpGs represented on the Illumina 450K array demonstrated little difference between these 2 compartments, those in the promoter region, including oestrogen responsive elements and NF-κB binding sites, revealed large differences owing to increased methylation levels in the proximal (compared with the fimbrial) compartment in mutation carriers (Fig. 3). This is expected of a polycomb repressor complex 2 target gene like *HOXC4* and agrees with previous data^18^,^19^. HOXC4 immunohistochemistry demonstrated significantly higher fimbrial compared to proximal staining in mutation carriers compared with controls (Fig. 4a).

### HOXC4-mediated AID expression

HOXC4 is a key trigger for expression of AID^20^,^21^. This enzyme plays an important role in antibody class switch DNA recombination, somatic hypermutation^22^, DNA methylome reprogramming, stem cell biology^23^,^24^ and in B-cell maturation by site-specific demethylation and diversification of the DNA methylome in germinal center B cells^25^. It may also be involved in epithelial carcinogenesis^26^.

Experiments with short term cultures of primary fimbrial fallopian tube cells (Supplementary Fig. 4) and with the SKOV3 ovarian cancer cell line demonstrated that NF-κB -activating inflammatory signals (that is, TNF-α and IL1-β), which are known to (i) be released in the pelvis under conditions known as risk factors for HGSEMCs (that is, pelvic inflammatory disease or frequent ovulation associated with the need for repeated repair of ovulatory defects in close proximity to the fimbrial tube) and (ii) be more abundant in women with a family history due to a *BRCA1* mutation^27^,^28^,^29^, lead to increased expression of AID (Fig. 4b). Reduction of HOXC4 levels (Supplementary Fig. 5) substantially reduced the AID-inducing effect of TNF-α and IL1-β (Fig. 4b). Consistent with HOXC4 (ref. 30) and AID^23^ function, HOXC4 increases the fraction of side population (stem and progenitor) cells (Fig. 4c, Supplementary Fig. 6). AID overexpression (Supplementary Fig. 7) enhances HOXC4 expression (Fig. 4d). Whereas AID expression was almost absent in the proximal fallopian tube, it was expressed at low levels in the fimbriae of control women but at high levels in fimbriae from *BRCA1/2* mutation carriers consistent with our *in vitro* data (Fig. 4e,f).

### Role of AID in fimbrial epithelium

**Figure 5:**
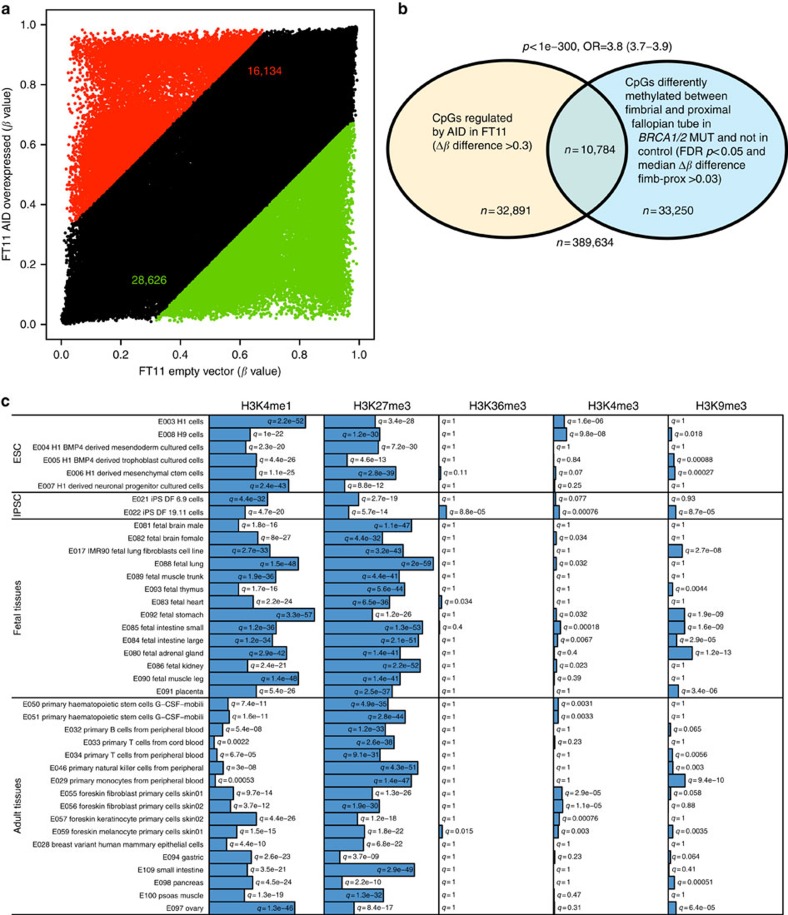
AID-mediated epigenetic reprogramming in primary cultures of fallopian tube epithelium. (**a**) Scatter-plot showing *β* values in AID-overexpressing FT11 cells compared with FT11 cells transfected with the empty vector. CpGs with Δ*β* >0.3 are coloured red and green respectively for hyper- and hypo-methylated CpGs in AID-overexpressing cells, with the numbers of CpGs categorized in this way indicated in these respective colours. (**b**) Venn-diagram illustrating significant overlap (with *p*-value calculated with Fisher's exact test) between CpGs that are differentially methylated between fimbrial and proximal fallopian tube compartments and in FT11 cells force-expressing AID. (**c**) eFORGE *q*-values (for explanation see Fig. 2) for overlap between the 1,000 CpGs showing the greatest hypomethylation differences between cells force-expressing AID and H3K4me1, H3K27me3, H3K36me3, H3K4me3 and H3K9me3 peaks from the Roadmap Epigenomics consortium (consolidated epigenomes release 2015, covering 39 tissues).

Forced-expression of AID led to a consistent increase in side population cells in agreement with AID's role in stem cell biology (Fig. 4g and Supplementary Fig. 8). Overexpression of AID in primary cultures derived from fimbrial cells triggered a substantial epigenetic reprogramming (Fig. 5a). As expected, a high number of CpGs (*n*=28,626) showed reduced methylation levels (>0.3 *β* difference of AID-overexpressing cells compared with empty vector controls, Supplementary Data 5). A smaller number of CpGs (*n*=16,134) showed an increase in DNA methylation (>0.3 *β* difference, Supplementary Data 6). This was confirmed—albeit to a smaller extent—in other cell lines (Supplementary Fig. 9). We observed a significant overlap between CpGs affected by AID in cultured fimbrial cells and those that are differentially methylated in fimbrial compared with proximal fallopian tube compartments in mutation carriers (Fig. 5b and Supplementary Fig. 10). It is particularly notable that regions marked with H3K4me1 or H3K27me in stem cells and fetal tissues are those with the highest enrichment score for overlap with regions hypomethylated (Fig. 5c and Supplementary Fig. 11) and hypermethylated upon AID overexpression (Supplementary Fig. 12). Remarkably, the adult tissue signature most enriched within the *in vitro* AID-induced epigenetic reprogramming model of cultured fimbrial cells was the ovary, exactly as seen in the *in vivo BRCA* fimbrial-proximal model (Fig. 2).

### Link between reprogramming and cancer

The data presented thus far are consistent with the view that AID induces epigenetic reprogramming resulting in a decreased state of cellular differentiation in the fimbriae of *BRCA* mutation carriers.

**Figure 6:**
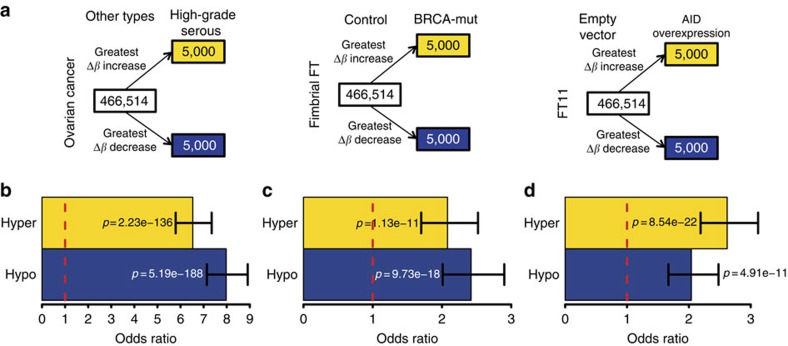
Preservation of AID-mediated reprogramming in high-grade serous cancers. (**a**, left) 5,000 CpGs with the greatest *β* increase and decrease in high-grade serous ovarian cancer (*n*=124) compared with non-serous ovarian cancer types (*n*=36) (clear cell, endometrioid and mucinous) were selected. (middle) 5,000 CpGs with the greatest median *β* increase and decrease in fimbrial compartments of *BRCA* mutation carriers compared with non-carriers were selected (called hyper- and hypomethylated respectively). (right) 5,000 CpGs with the greatest *β* increase and decrease in primary cultures of FT11 cells force-expressing AID compared with cells transfected with an empty vector were selected. (**b**) The 5,000 most hypermethylated and 5,000 most hypomethylated CpGs in fimbrial (*BRCA* mutation carriers versus control) were tested for overlap with the 5,000 CpGs showing the greatest *β* increase and decrease, respectively, in high-grade serous carcinomas compared with non-serous subtypes (clear cell, endometrioid and mucinous). (**c**) The 5,000 CpGs showing the greatest *β* increase and decrease in serous carcinomas compared with other cancer types were tested for overlap with those 5,000 CpGs showing the biggest increase and decrease in methylation, respectively, in cultured cells force-expressing AID compared with cells transfected with empty vector. (**d**) The 5,000 most hyper- and hypomethylated CpGs in fimbrial samples (*BRCA* mutation carriers versus control) were similarly tested for overlap with the 5,000 CpGs with greatest *β* increase and decrease, respectively, in the cultured cells force-expressing AID. All *p*-values were derived using Fisher's exact test; all error bars indicate 95% confidence intervals on the estimated odds ratios.

To further substantiate the idea that reprogrammed CpG sites in histologically normal fimbriae of *BRCA* mutation carriers are relevant to cancer development and biology, we hypothesized that the same CpGs should be enriched amongst those differentially methylated between HGSEMCs (by far most prevalent histological subtype of ovarian cancer in mutation carriers) and the other extra-uterine Müllerian cancer subtypes. The 5,000 CpGs (that is, approximately the top 1% available on the array) with greatest increase or decrease in methylation level between *BRCA* mutation carriers and controls in the fimbrial compartment (Fig. 6a) overlapped very significantly with corresponding HGSEMC-specific CpGs (Fig. 6b). The finding of a highly significant overlap between CpGs that are modulated by AID expression in primary fimbrial cells and those discriminating HGSEMCs from non-serous subtypes (Fig. 6c and Supplementary Fig. 13) further attests to AID's key role in triggering epigenetic reprogramming in tissues at increased risk of HGSEMC in *BRCA* mutation carriers. CpGs whose methylation levels increase or decrease in the fimbriae in *BRCA* mutation carriers compared with controls strongly overlap with those regulated by AID in cell lines (Fig. 6d and Supplementary Fig. 14). The results are very similar when selecting those CpGs whose methylation levels increase or decrease in fimbrial compared with proximal fallopian tube in *BRCA* mutation carriers but not in controls (Supplementary Fig. 15). The extent of methylation overlap between serous subtypes of extra-uterine Müllerian cancers and the fimbrial compartment of individuals with familial predisposition to these tumours characterized by increased AID expression underscores the role of epigenetic reprogramming, in particular AID-mediated hypomethylation, at the earliest stages of cancer development in *BRCA* mutation carriers.

## Discussion

During the past decade, clear evidence has accumulated that epigenetic programming determines cell fate and that these programs at all levels of the epigenome—including DNA methylation—are dramatically different in established cancers compared with the tissues from which they originate^11^,^31^. Naturally in humans it is almost impossible to assess when epigenetic reprogramming is initiated in the progression towards cancer development. Studying this phenomenon is complicated further by the fact that epigenetic reprogramming is (i) tissue-specific and (ii) subject to alteration by age and various environmental exposures. The human fallopian tube is one of the very few human organs which allow the study of true pre-neoplastic reprogramming events because ∼40% of *BRCA* mutation carriers are eventually diagnosed with a HGSEMC originating in the fimbrial end of the fallopian tube, providing an opportunity to examine tissues at elevated cancer risk before morphological evidence of neoplastic transformation. In addition, epigenetic background noise triggered by factors unrelated to cancer development can be adjusted for by analysing the proximal end of the fallopian tube, which is not associated with increased cancer risk. Both our descriptive and functional data demonstrate that AID-mediated reprogramming is a prevalent and early phenomenon in the fimbriae of *BRCA* mutation carriers. This epigenetic reprogramming largely resets tissue-specific programs seen in fetal and undifferentiated (stem) cells. Importantly, the observation that these alterations are preserved in high-grade serous compared with non-serous cancers, which do not originate in the fallopian tube, adds further evidence to the functional importance of early epigenetic reprogramming.

*BRCA1/2* germline mutation carriers have an elevated cancer risk restricted to breast and reproductive organs in spite of the fact that the encoded proteins are expressed in nearly every tissue. Both BRCA1 and BRCA2 have important roles in chromosome duplication, maintenance, and segregation across the cell cycle and are regarded as the ‘chromosome custodians'^7^. Hence if this cell-autonomous effect was the only driver of neoplastic transformation in *BRCA* mutation carriers, organs with both a high cell number and cell turnover should be most susceptible to cancerous transformation. The fact that *BRCA* mutation carriers do not have an elevated risk of cancer in organs with high cell turnover, for example the colon or the bone marrow, underscores the importance of understanding systemic cell-non-autonomous mechanisms in triggering early events, including epigenetic events, in *BRCA*-driven cancer development.

The fact that constitutive and ubiquitous expression of AID in transgenic mice leads to cancer development very early on in various organs^32^,^33^ leading to early death^33^ supports the idea of AID being an essential component of carcinogenesis. Currently we can only speculate as to why AID is overexpressed in *BRCA1/2* mutation carriers. In the setting of gastric cancer, exogenous factors like *Helicobacter pylori* trigger an aberrant expression of AID in gastric cells which leads to an accumulation of nucleotide alterations in the TP53 tumour suppressor gene^26^ and therefore highlights the relevance of cell-‘non-autonomous' factors in driving AID expression. In the *BRCA* mutation carrier setting, cell ‘non-autonomous' factors such as aberrant levels and/or dynamics of cytokines released from the ovary during the repair of the ovarian surface after each monthly ovulation, which affect the fimbriae in a paracrine fashion, or a higher rate of ascending infections, may drive cancer predisposing aberrant AID expression. Alternatively, cell ‘non-autonomous' factors known to be strongly associated with HGSEMC risk (that is, high number of ovulations and pelvic inflammatory disease^34^) could lead to aberrantly high AID. Some, although rather preliminary, support for a cell ‘non-autonomous' mechanism comes from the observation that a *BRCA1* mutation enhances Interleukin-1β expression in ovarian surface epithelial cells^29^, and that *BRCA* mutation carriers' associated epigenetic mis-programming of immune cells^18^ may lead to subtle immune defects which in turn facilitate microbial ascension from the vagina to the fallopian tube. Additional evidence for a cell ‘non-autonomous' factor comes from our previous observation that *BRCA* mutation carriers demonstrate higher oestrogen levels in the luteal phase^35^. As indicated above, after ovulation during the luteal phase, there is high inflammatory activity in close proximity to the fimbriae, and estrogen—via activation of HOXC4—might further increase AID expression in *BRCA* carriers during this stage of the menstrual cycle. Eventually, AID-mediated reprogramming events could then accumulate at the fimbriae.

Recently we demonstrated the existence of widespread epigenetic field defects in normal tissues adjacent to breast cancers, with these defects becoming enriched during breast cancer progression^36^. Here we confirmed that the same principle—existence of DNAme changes before cancer formation—applies also to other cancers.

In summary, we demonstrated that epigenetic reprogramming in morphologically normal fimbrial cells of *BRCA1/2* mutation carriers, which have a high risk for neoplastic transformation, is highly prevalent and mediated by AID, and that the changes in methylation are maintained in HGSEMCs. These findings open entirely new windows of opportunities to develop novel strategies for non-invasive cancer-preventive measures.

## Methods

### Patient cohort description

Based on our previous study where we analysed blood cells of 72 *BRCA1/2* mutation carriers and 72 controls^18^, we estimated that 50–60 mutation carriers and controls should provide sufficient power to detect methylation differences (between fimbrial at elevated cancer risk and proximal at average cancer risk fallopian tubes) between carriers and controls. The study and the use of archived samples has been approved by the UCL/University College London Hospitals (UCLH) Biobank for Studying Health and Disease Ethical Review Committee under REC 10/H1306/42 (EC08.12: Retrospective study of fallopian tubes and ovaries from women with *BRCA1/2* mutations) did not require informed consent.

After removing samples in our quality control procedure, we analysed DNAme data from 59 controls, 30 *BRCA1* mutation carriers and 26 *BRCA2* mutation carriers. Of the controls and the mutation carriers, 49 and 52, respectively, had data available for both fimbrial and proximal ends of the fallopian tube. The median age of controls was 50 years (range 15 to 81), the median age of *BRCA1* mutation carriers was 46.5 years (range 36–66), and the median age of *BRCA2* mutation carriers was 50.5 years (range 40–65). There was no significant age difference between *BRCA1/2* mutation carriers and controls (*p*=0.66, Wilcox-test). The controls had an operation due to one of the following conditions: fibroids (*n*=20), adenomyosis (*n*=10), cervical pathology (*n*=4), uterine prolapse (*n*=4), polyps (*n*=4) and other (*n*=17).

We also analysed cancer samples of various histological subtypes^37^: (i) 124 high-grade serous samples, of which 115 were stages 3–4 (median age was 58 years, range 36–84) and 36 other cancer subtypes, of which (ii) 17 were endometrioid, (iii) 13 were clear cell and (iv) six were mucinous. Out of the 36 non-serous subtypes, 19 were stages 3–4. Median age of these 36 patients with other histologies was 56.5 years (range 29–85).

### DNA extraction and DNA methylation analyses

Formalin fixed paraffin-embedded (FFPE) tissue blocks were retrieved from UCL Biobank (NC09.13). Histopathological features of fimbrial and proximal compartment of fallopian tube from *BRCA* carriers and controls were carefully examined. Samples negative for serous tubal intraepithelial carcinoma lesions were chosen for DNA isolation to characterize pre-cancerous epigenetic changes. For DNA isolation, a core of 3 × 0.6 mm was taken from each block representing fimbrial and proximal end of fallopian tubes from both *BRCA* carriers and matched controls. The DNA was isolated using QIAamp DNA FFPE Tissue Kit as per manufacturer's protocol with minor modifications (Dewaxing for 4 h in xylene and proteinase digestion performed overnight, other procedures were as per the instructions). DNA from cell lines (untreated, vector control and AID-overexpressing cells) was isolated using AllPrep DNA/RNA Mini Kit (Qiagen) as per manufacturers instruction. DNA was quantified using Nandrop and restored using the Infinium FFPE DNA Restore Kit and then 200 ng of DNA was bisulfite converted using the EZ DNA Methylation-Gold and subjected to methylation analysis on the Illumina Infinium Human Methylation450 BeadChip.

For cancer samples we used frozen tissue. DNA was isolated from tissue samples using the Qiagen DNeasy Blood and Tissue Kit (Qiagen Ltd, UK, 69506) and 600 ng was bisulfite converted using the Zymo methylation Kits (Zymo Research Inc, USA, D5004/8). Genome wide methylation analysis was performed using the Illumina Infinium Human Methylation450 BeadChip. Fallopian tube data are available from the Gene Expression Omnibus (GEO) under accession number GSE74845 and ovarian cancer data are available under accession number GSE72021.

### Cell culture

Established cancer cell lines (SKOV3 and HOC7 from ATCC #HTB77 and from Ronald Buick at University of Toronto^38^, respectively). The cells were maintained in DMEM/F-12 supplemented with 10% fetal bovine serum and 1% penicillin-streptomycin (Life Technologies). Cells were seeded into six-well tissue culture plates for cytokine (TNFα and IL1B) treatment or lentivirus infection. The cell lines tested negative for mycoplasma contamination.

### Primary fallopian tube secretory epithelial cell culture

Patients undergoing salpingectomy with hysterectomy for endometrial cancer or reasons other than serous cancer at UCLH were consented to donate fallopian tube tissue surplus to diagnostic requirements (that is, not needed for clinical care) following UCL ethical guidelines (women provided written informed consents and samples were collected under the NRES Committee London—Surrey Borders Research Ethics Committee approval; 14/LO/1633). Fimbrial fallopian tube secretory epithelial cells were isolated and cultured as described by Karst and Drapkin^39^. Briefly, fimbrial tissues were carefullly disected out by an experienced pathologist, macerated and digested in a dissociation medium (0.14% pronase and 0.01% DNase in MEM) for 36 h at 4 °C. Cells were harvested by centrifugation and incubated at 37 °C on collagen coated six-well (35 mm) tissue culture plates in DMEM/F-12 supplimented with 2% Ultroser G (Pall corporation, France) and 1% penicillin-streptomycin. Adherent cells at∼50% confluence were immortalized by infection with retroviral constructs for hTERT stable expression. Cells were phenotyped: firstly by determining mRNA expression of PAX8 (Mullerian marker) and Cytokeratin 7 (CK7, epithelial marker) using quantitative PCR; and secondly by immunofluorescent staining. Cells were seeded into 35 mm tissue culture plates for subsequent experiments. All experiments were carried out before cells started to senesce. Cell senescence was a major hurdle for long-term incubation studies, but primary fimbrial cells offer the advantage of mimicking biological events close to ovarian cancer studies.

### Stable cell lines

293T cells were cultured into T-75 tissue culture flasks for 1 day before transfection. Complexes of packaging plasmid (7.5 μg, pCMV-dR8.2 dvp or p8.91), envelope plasmid (2.5 μg pCMV-VSV-G or pMD.2G) and construct (10 μg; as shown in Supplementary Table 1) were prepared using lipofectamine 2000 (Life technologies) as per manufacturer's instructions (third Generation Lenti-Combo Packing Mix was used for lentivirual production when constructs from). 293T cells at 70% confluence were transfected with complexes prepared as described above. The culture medium was changed 24 h post transfection and virus was harvested at 48 and 72 h post transfection. Virus was concentrated using Lenti-X concentrator (Clonetech) as per manufacturers instructions. Cells (HOC7, SKOV3 and primary fimbrial cells) were cultured into 35 mm well plates and infected with lentiviral constructs in presence of polybrene (1 μg ml^−1^) (for HOXC4 or AID overexpression, shRNA against HOXC4 or corresponding empty vector) or retroviral constructs (hTERT overexpression). Cells stably carrying virus-mediated gene integration were selected by treating with puromycin (1 μg ml^−1^) and maintained in puromycin medium at least for a week or until all the untreated control cells were dead. Selective overexpression or suppression of genes of interest was measured by real-time PCR. For HOXC4 knockdown experiments, different constructs were screened, and the construct showing maximum gene knockdown efficiency was used for cytokine treatment experiments.

### RNA isolation and gene expression analysis

Total RNA was isolated using RNeasy Mini Kit (Qiagen) as per manufacturer's recommendation. RNA was eluted in water and quantified by ultraviolet absorbance measurements with a nanodrop instrument. One microgram of total RNA was used for cDNA synthesis using High-Capacity cDNA Reverse Transcription Kit (Applied Biosystems) in a volume of 20 μl as per manufacturer's instructions. Gene expression was quantified using real-time PCR. The reaction mixture consisted of 25 μl of × 2 Power SYBR Green master mix, 1 μl of 10 μM forward and reverse primer each (primer sequence provided in Supplementary Table 2), 22 μl water and 1 μl cDNA (50 ng) in a final reaction volume of 50 μl, in triplicates. Real-time PCR was performed using Eppendorf Mastercycler RealPlex 2. Relative gene expression was analysed using ΔΔCt methods using GAPDH as endogenous control for normalization. Relative fold changes in expression levels are represented.

### Flow cytometry analysis of side population

Actively growing cells were trypsinised and resuspended in growth medium at 10^6^ cells per ml. One millilitre of cell suspension was treated with Hoechst 33342 (5 μg ml^−1^) for 90 min at 37 °C. Cells were pelleted and resuspended in ice-cold PBS with 2% fetal bovine serum and propidium iodide (2 μg ml^−1^). Live cells were analysed by flow cytometry (BD LSRII); cells with low Hoechst blue and red staining were considered for side population analysis.

### Immunohistochemistry

All reagents other than primary antibodies were from Leica Biosystems (Newcastle-Upon-Tyne, UK) and staining procedures were performed using Leica Bond-III. For immunohistochemical analysis of protein expression, 3 mm paraffin sections from fimbrial and proximal end of fallopian tube (HOXC4 and AID expression) were treated for automated dewaxing (Leica Bond Dewax AR9222). Automated antigen retrieval was then performed on the sections. Citrate based, pH 6.0 Leica Bond ER1 (HOXC4) or Tris-EDTA based, pH 9.0 Leica Bond ER2 (AID) was applied to the slides before heating to 100 °C (30 min for HOXC4 and 20 min for AID). Primary antibodies against HOXC4 (dilution 1/30) or AID (1/3,800) were applied to the respective slides with 15 min incubations. Endogenous peroxidase was blocked using 3–4% (v/v) hydrogen peroxide (Part of Leica Bond Refine Polymer Kit, DS9800). An anti-rabbit secondary antibody (Bond Post Primary, part of the Leica Bond Refine Polymer Kit, DS9800) was applied for 8 min for HOXC4. For AID, an anti-rat secondary reagent (Vector Laboratories cat # AI-4001, diluted 1/100) was applied for 15 min. Signal visualization was performed using Bond Polymer (Part of Polymer Refine Polymer Kit, cat # DS9800, HRP based) for 8 min for all antibodies. DAB was applied for 10 min for all slides, then Leica Bond DAB enhancer (copper sulfate, concentration proprietary) was applied for 5 min. Cell nuclei were counterstained with haematoxylin, then dehydrated and mounted in DPX-based mountant. The Leica Bond Polymer Detection Kit (DS9800) was used for peroxidase blocking, visualization and counterstaining. Bond Wash (AR9590) and deionized water were used for washing steps between reagent steps. AID and HOXC4 staining was observed at the fimbrial and proximal ends of the same fallopian tubes, among controls and *BRCA* mutation carriers. Relative intensity of AID or HOXC4 staining was scored by experienced pathologists (RA and JM). Scores for cytoplasmic and nuclear intensity (on a scale of 1–3) times percentage of positive cells were derived independently for the proximal and the fimbrial fallopian tube regions. The scoring was done by two independent pathologists blinded for the *BRCA* status.

### Immunofluorescence staining

Primary fimbrial secretory epithelial cells were cultured on coverslips in six-well plates. Cells were washed three times with PBS and fixed with 4% paraformaldehyde at room temperature for 20 min. Cells were permealized using 0.5% Triton-X 100. Blocking was done using bovine serum albumin at room temperature for 1 h. Cells were treated with primary antibodies for PAX8 (Source Biosceince, Rabbit polyclonal, 1/1,000) or Cytokeratin 7 (Rabbit monoclonal antibody- EPR1619Y; Abcam, catalogue: ab68459; dilution: 1/500) diluted in blocking buffer and incubated at 4 °C overnight. Cells were washed with PBST, and stained with secondary antibody (Fluorescein Goat Anti-Rabbit IgG Antibody; Victor laboratories, catalogue: FI-1000, 10 μg ml^−1^) for 1 h at room temperature. Nuclei were counterstained with Hoechst 33352 (2 μM) and observed under an inverted microscope. Washing between steps was carried out using PBST.

### Data-processing and analysis

DNA methylation data were obtained via the Illumina HumanMethylation 450 K array, relating to healthy fimbrial and proximal tissue samples as well as the cell line experiments outlined above. In addition, ovarian cancer samples were incorporated into the analyses illustrated in Fig. 6 and Supplementary Figs 13 and 15. Fallopian tube data are available from the Gene Expression Omnibus (GEO) under accession number GSE74845 and ovarian cancer data are available under accession number GSE72021. All methylation data were background-corrected, without further normalization. Samples were removed if they had < 80% coverage across probes, and probes were subsequently removed if they had <95% coverage across samples. Any remaining probes with detection *p*-value >0.05 were replaced by KNN imputation, with *k*=5. We note that under testing with the additional data-processing steps of removal of the 93,382 cross-reactive/polymorphic 450k probes of Chen *et al*.^40^, and normalization with the SWAN or BMIQ methods, all results and their overall significance levels were unchanged.

*P*-values were obtained quantifying the observed methylation differences between fimbrial and proximal samples using LIMMA (linear modelling for microarray experiments)^41^. This was carried out separately for *BRCA1/2* MUT and control samples, and histograms of these *p*-values appear in Fig. 1b. On the basis of these tests, lists of significant CpGs in *BRCA1/2* MUT and control were identified according to FDR corrected *p*<0.05. The difference between these lists provides a list of CpGs significant in *BRCA1/2* MUT and not in control samples.

CpGs were then further selected as significant only when they corresponded to Δ*β* greater than a set threshold. For fimb-prox (*BRCA1/2* MUT) comparisons, this threshold was set at median Δ*β*>0.03 (with the median calculated across all *BRCA1/2* MUT samples), these CpGs appear in Supplementary Data 1 and 2. We expanded on this choice of threshold of 0.03, as follows: There is heterogeneity of DNA methylation at a given CpG locus in a given anatomical region (that is, fimbrial or proximal tube) within a given volunteer, and from volunteer to volunteer; by selecting all CpGs above this threshold median Δ*β* of 0.03 we found a large number of CpGs showing a much greater methylation change than 0.03 for many volunteers; specifically, of those CpGs selected with median Δ*β* > 0.03, on average 40% of volunteers showed Δ*β* >0.1, and 13% show Δ*β* >0.2; for the cell lines, the threshold was set as Δ*β* (AID overexpression—empty vector) >0.3 in FT11 (these CpGs are shown in Supplementary Data 5 and 6), 0.1 in FT8, 0.07 in FT7, 0.15 in HOC7 and 0.07 in SKOV3.

Genes were then identified as significant in fimb-prox and in the cell lines by carrying out a binomial test for each gene, at a significance level of FDR corrected *p*<0.05. These tests were carried out separately for hypermethylated and hypomethylated CpGs, leading to two lists of significant genes. Also based on these lists of significant genes, GSEA was carried out, as follows: A list of 6,811 gene set definitions, downloaded from the Broad Institute Molecular Signatures Database (http://www.broadinstitute.org), were tested one by one for enrichment by the lists of significant genes; these enrichment tests were carried out using one-sided Fisher's exact tests, and gene-sets were identified as significant according to FDR *p*<0.05. These GSEA results appear in Supplementary Data 3 and 4.

The significance *p*-values shown in Fig. 4 were calculated as follows: The log of the expression levels in (b) and (d) were assumed to follow a normal distribution, and hence two-sided *t*-tests based on logs of the plotted values were used; the count-data on which the percentages shown in (c) and (g) are based were assumed to follow a Poisson distribution with a mean great enough to make a Gaussian approximation, and hence *t*-tests were again used. The data shown in (a) and (e) were assumed to be drawn from an unknown distribution and hence two-sided Wilcox tests were used.

### eFORGE analyses

eFORGE (http://eforge.cs.ucl.ac.uk/) is a tool for identifying overlap enrichment of EWAS regions with regulatory elements marked by DNase I Hypersensitive Sites (DHSs) across different cell types and tissues. In addition to ENCODE and Roadmap Epigenomics DHS data, eFORGE also includes histone mark data (H3K4me1, H3K27me3, H3K36me3, H3K4me3 and H3K9me3) from the Roadmap Epigenomics Consortium 2015 data release. The software is freely available from: https://github.com/charlesbreeze/eFORGE.

eFORGE analysis for this study focused on (a) CpGs discriminating fimbrial from proximal samples (fimb-prox), and on (b) CpGs distinguishing AID-overexpressing FT11 cells from empty vector controls. Analysis was performed on the top 1,000 hypo- and hypermethylated CpGs in (a) and (b), generating 1,000 random 450k background sets for each input list, selecting background probes with similar gene and CpG Island annotation. eFORGE analysis was also performed on CpGs hypo- and hypermethylated upon AID overexpression in FT7, FT8, HOC7 and SKOV3 cells.

### Data availability

The DNA methylation microarray data from human tissues that support the findings of this study have been deposited in Gene Expression Omnibus (GEO) data repository under accession codes GSE74845 and GSE72021.

## Additional information

**Accession codes:** The DNA methylation microarray data has been deposited in the Gene Expression Omnibus (GEO) database under accession codes GSE74845 and GSE72021.

**How to cite this article:** Bartlett, T. E. *et al*. Epigenetic reprogramming of fallopian tube fimbriae in *BRCA* mutation carriers defines early ovarian cancer evolution. *Nat. Commun.* 7:11620 doi: 10.1038/ncomms11620 (2016).

## Supplementary Material

Supplementary Information Supplementary Figures 1-15 and Supplementary Tables 1-2

Supplementary Data 1 Top CpGs which are hypermethylated in fimbrial compared to proximal tube in BRCA1/2 mutation carriers but not in controls. CpGs are included with median delta-beta fimb-prox greater than 0.03, and FDR-corrected p<0.05. Genomic information is also provided for each CpG.

Supplementary Data 2 Top CpGs which are hypomethylated in fimbrial compared to proximal tube in BRCA1/2 mutation carriers but not in controls. CpGs are included with median delta-beta fimb-prox greater than 0.03, and FDR-corrected p<0.05. Genomic information is also provided for each CpG.

Supplementary Data 3 Gene-set enrichment analysis of those CpGs which are hypermethylated in fimbrial compared to proximal tube in BRCA1/2 mutation carriers but not in controls. CpGs with median delta beta >0.03 are included in the analysis (7421 CpGs). Odds-ratios and Fisher-test p-values quantify enrichment by specified Broad-institute gene-groups by genes identified as significant according to their corresponding numbers of significant hypermethylated CpGs.

Supplementary Data 4 Gene-set enrichment analysis of those CpGs which are hypomethylated in fimbrial compared to proximal tube in BRCA1/2 mutation carriers but not in controls. CpGs with median delta beta >0.03 are included in the analysis (36977 CpGs). Odds-ratios and Fisher-test p-values quantify enrichment by specified Broad-institute gene-groups by genes identified as significant according to their corresponding numbers of significant hypomethylated CpGs.

Supplementary Data 5 Top CpGs which are hypomethylated in FT11 AID overexpression cells compared to empty vector. CpGs are included with delta-beta greater than 0.3. Genomic information is also provided for each CpG.

Supplementary Data 6 Top CpGs which are hypermethylated in FT11 AID overexpression cells compared to empty vector. CpGs are included with delta-beta greater than 0.3. Genomic information is also provided for each CpG.
