# Supplementary material for: Epigenetic reprogramming of fallopian tube fimbriae in BRCA mutation carriers defines early ovarian cancer evolution
Source: Nat Commun. 2016 May 24;7:11620. doi: 10.1038/ncomms11620 (PMC4890182; doi:10.1038/ncomms11620)
Supplement: Supplementary Information — Supplementary Figures 1-15 and Supplementary Tables 1-2 [file ncomms11620-s1.pdf]

SUPPLEMENTARY FIGURES:

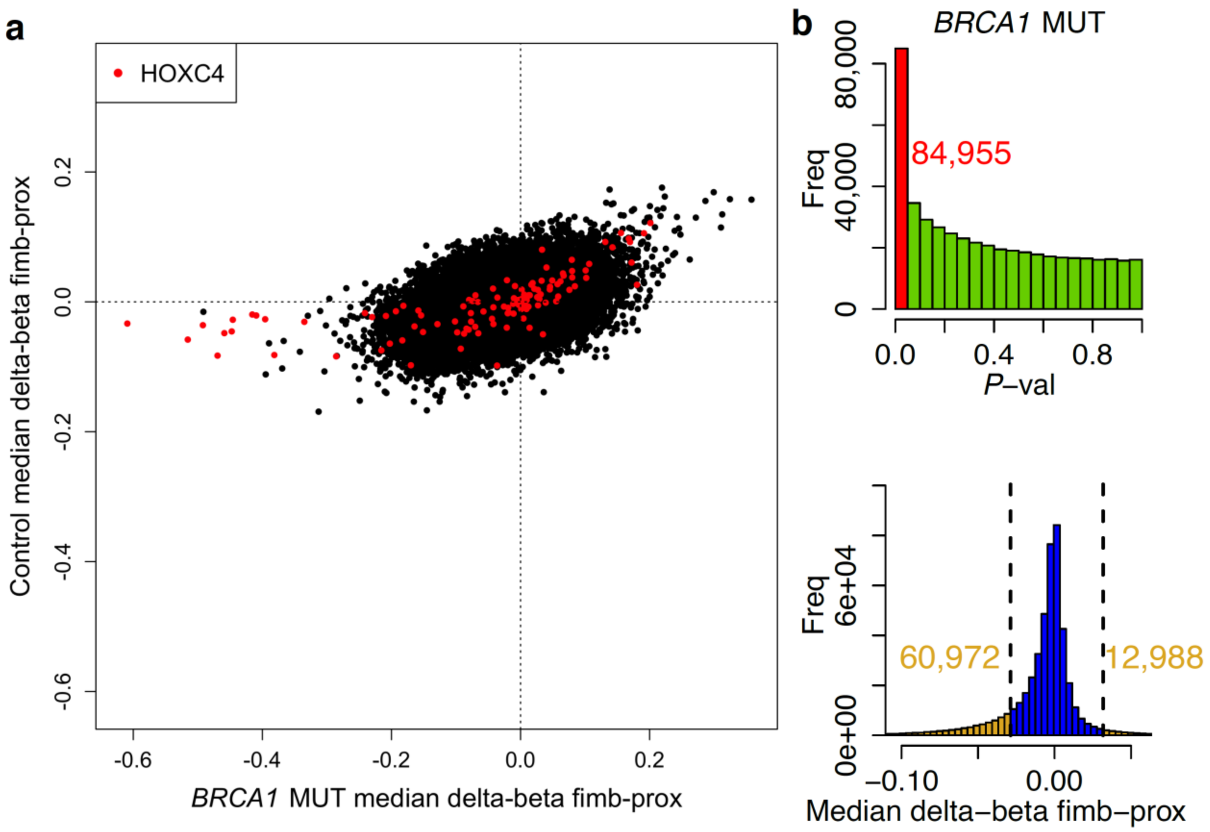

Supplementary Figure 1. Differences in the methylation profile of the proximal *versus* fimbrial compartments of the Fallopian tube in *BRCA1* mutation carriers and controls (a) Scatter-plot showing delta-beta fimb-prox in controls (*n*=49) against *BRCA1* mutation carriers (*n*=29). (b) *T*-test *p*-value histograms show concentrations of significant CpGs discriminating fimbrial from proximal *BRCA1* mutation carrier samples.

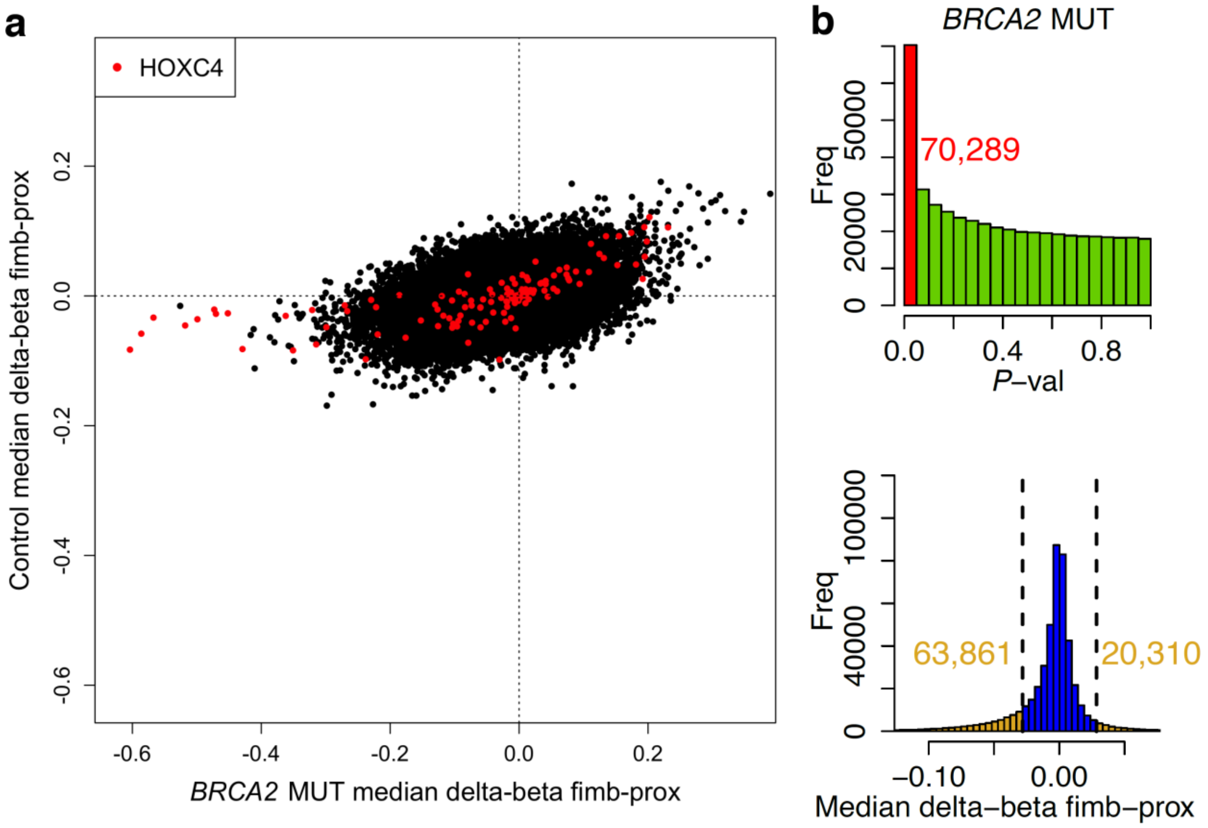

Supplementary Figure 2. Differences in the methylation profile of the proximal *versus* fimbrial compartments of the Fallopian tube in *BRCA2* mutation carriers and controls
(a) Scatter-plot showing delta-beta fimb-prox in controls (n=49) against *BRCA2* mutation carriers (n=23). (b) *T*-test *p*-value histograms show concentrations of significant CpGs discriminating fimbrial from proximal *BRCA2* mutation carrier samples.

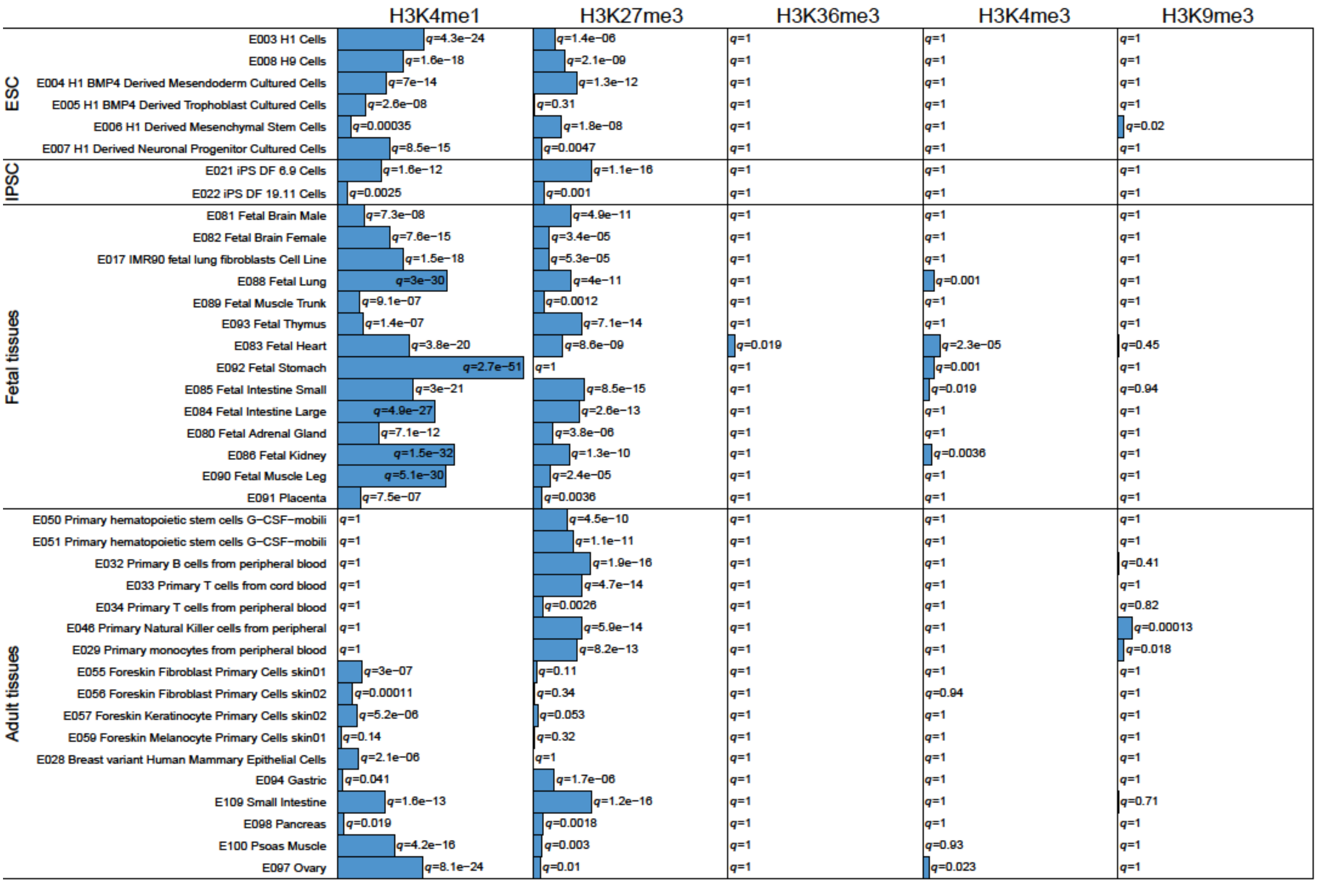

Supplementary Figure 3. Overlap with tissue specific patterns of histone modification

eFORGE *q*-values for enrichment of overlap for 1000 top hypermethylated fimb-prox CpGs with H3K4me1, H3K27me3, H3K36me3, H3K4me3 and H3K9me3 peaks from the Roadmap Epigenomics consortium (consolidated epigenomes release 2015, covering 39 tissues). For each set of differentially methylated positions (DMPs), an overlap analysis is performed against the functional elements from either data source for each cell sample separately (ENCODE and Roadmap), and the number of overlaps is counted. A background distribution of the expected overlap counts for this DMP set is obtained by picking sets of the same number of DMPs as the test DMP set, matched for gene relationship and CpG island relationship annotation. The matched background sets are then overlapped with the functional elements and the background distribution of overlaps determined. By default 1000 matched sets are used. The enrichment value for the test DMP set is plotted as the -log10 (binomial *p*-value). The Benjamini-Yekutieli (BY) multiple-testing corrected *q*-value is evaluated to mark enrichments as significant at *q*<0.05.

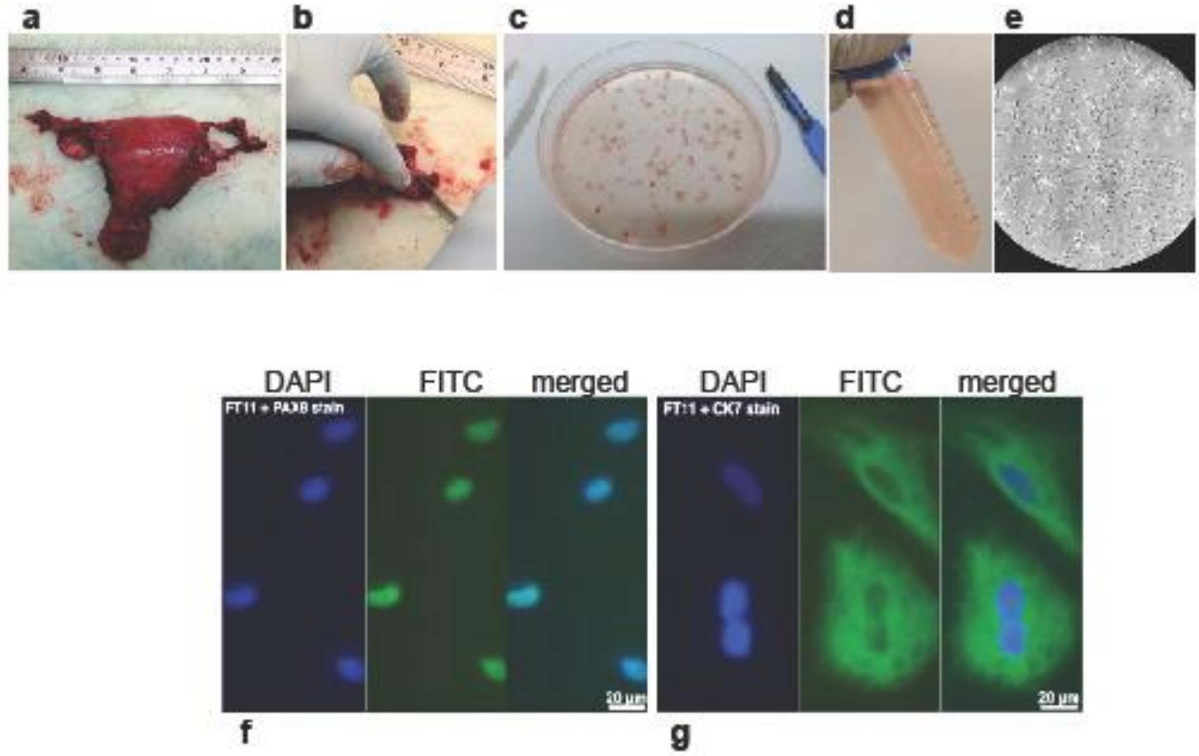

Supplementary Figure 4. Generation of primary fimbrial secretory epithelial cell culture

Fimbrial tissue (a) was macerated (b) and digested with pronase and DNase cocktail (c) for 36 hours at 4 °C (d). Cells were harvested by centrifugation and incubated at 37°C on collagen-coated tissue culture plates. Floating ciliary cells were washed away and adherent fimbrial secretory epithelial cells (e) were infected with a retroviral vector for hTERT. Fimbrial secretory epithelial cells were characterised by analysing expression of nuclear PAX8 (f) and cytoplasmic cytokeratin 7 (g) by immunofluorescence staining. Also, expression was confirmed by PCR (data not shown).

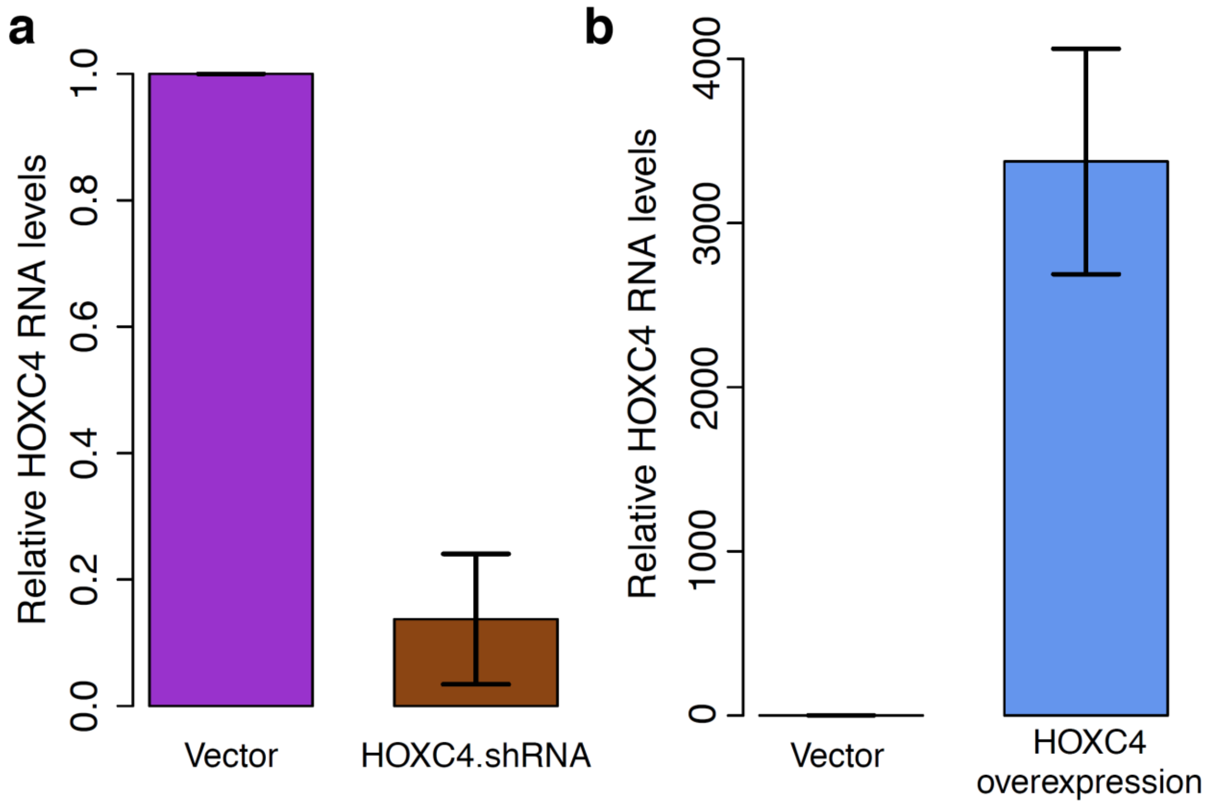

Supplementary Figure 5. **Modulation of HOXC4 expression in SKOV3 cells**

SKOV3 cells were infected with a lentiviral shRNA construct for stable cell suppression of HOXC4 (a) or a lentiviral construct for constitutive HOXC4 overexpression (b) (data from three independent experiments). Stable cell line was generated using puromycin selection and HOXC4 expression was analysed by real time PCR and relative expression is represented after normalization with GAPDH. Data were analysed using the delta delta Ct method. The error bars indicate one standard deviation.

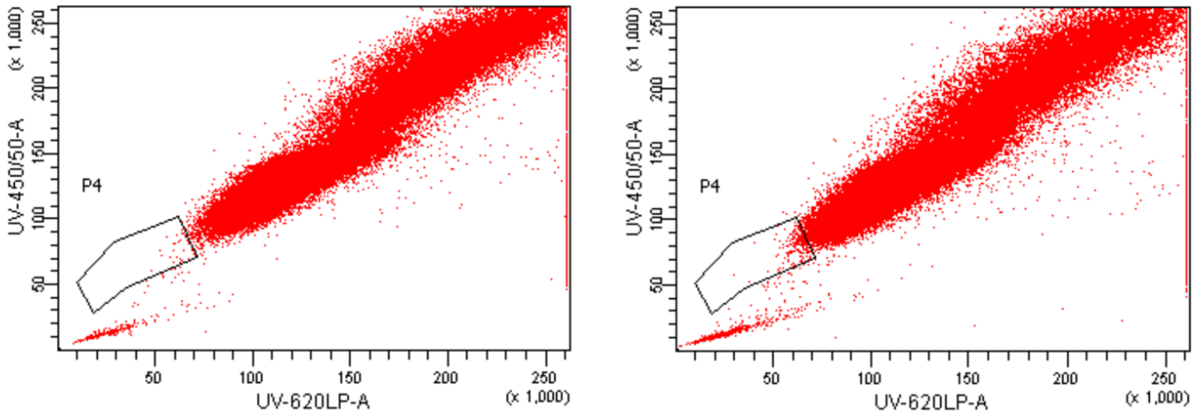

Vector **HOXC4 overexpression**

Supplementary Figure 6. **HOXC4 mediated enrichment of side population**

SKOV3 cells stable overexpressing HOXC4 were stained with Hoechst 3342 (5 µg/ml) for 90 minutes at 37 °C and counter stained with propidium iodide (2 µg/ml). Side population (stem like cells) with low Hoechst staining were analysed by flow cytometry. Side population is marked by gate P4.

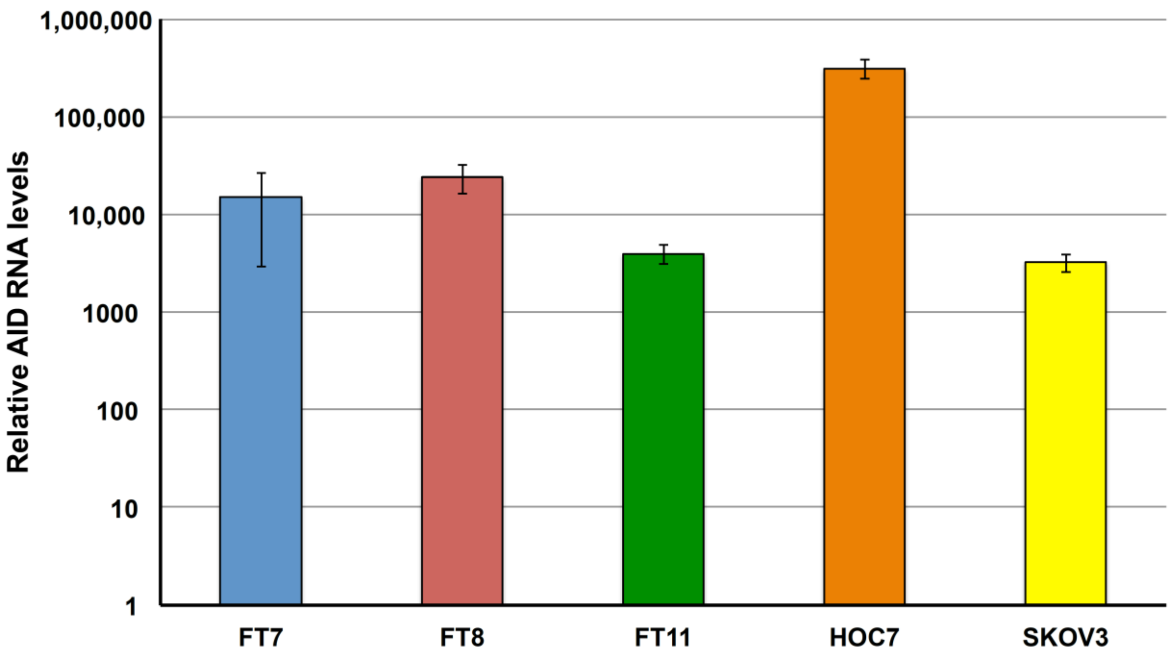

Supplementary Figure 7. **Modulation of AID expression**

Primary fimbrial secretory epithelial cells (FT7, FT8 and FT11), HOC7 and SKOV3 cells were infected with a lentiviral construct for constitutive, forced-expression of AID. AID expression was confirmed by real time RT-PCR using GAPDH as internal control. Data was analysed by the delta Ct method. Relative AID expression levels in AID over-expressing cells are shown as fold expression levels based on cells carrying the empty vector. Note: HOC7 cells carrying empty vector do not express any detectable AID after 45 cycles of amplification, Ct value of 40 was taken for HOC7 empty vector cells for calculating relative AID expression values. Data are from 3 independent experiments. Error bars: one standard deviation.

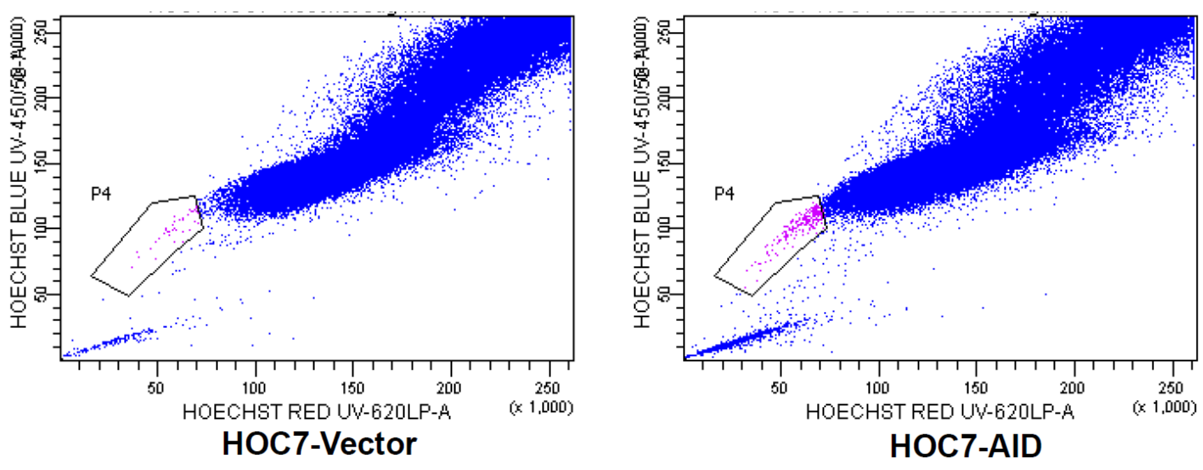

Supplementary Figure 8. **AID mediated enrichment of side population**

HOC7 cells stable overexpressing AID were stained with Hoechst 3342 (5 µg/ml) for 90 minutes at 37 °C and counter stained with propidium iodide (2 µg/ml). Side population (stem like cells) with low Hoechst staining were analysed by flow cytometry. Side population is marked by gate P4.

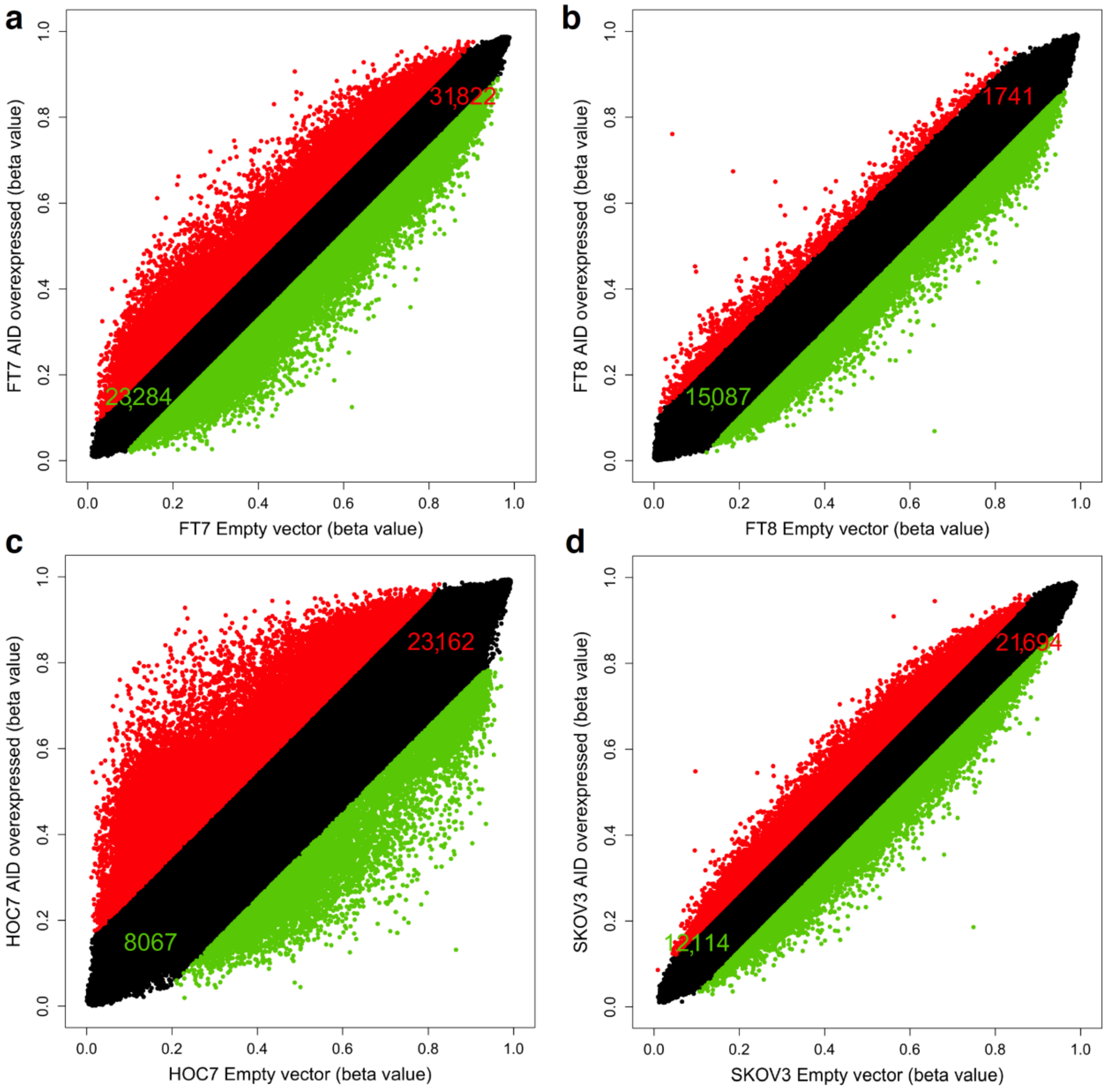

Supplementary Figure 9. Scatter-plots showing beta-values in AID-overexpressing cells against those transfected with empty vector

CpGs with delta-beta greater than the threshold are coloured red and green respectively for hypermethylated and hypo-methylated, with the numbers of CpGs categorised in this way indicated in these respective colours. (A) FT7, delta-beta threshold = 0.07. (B) FT8, delta-beta threshold = 0.1. (C) HOC7, delta-beta threshold = 0.15. (D) SKOV3, delta-beta threshold = 0.07.

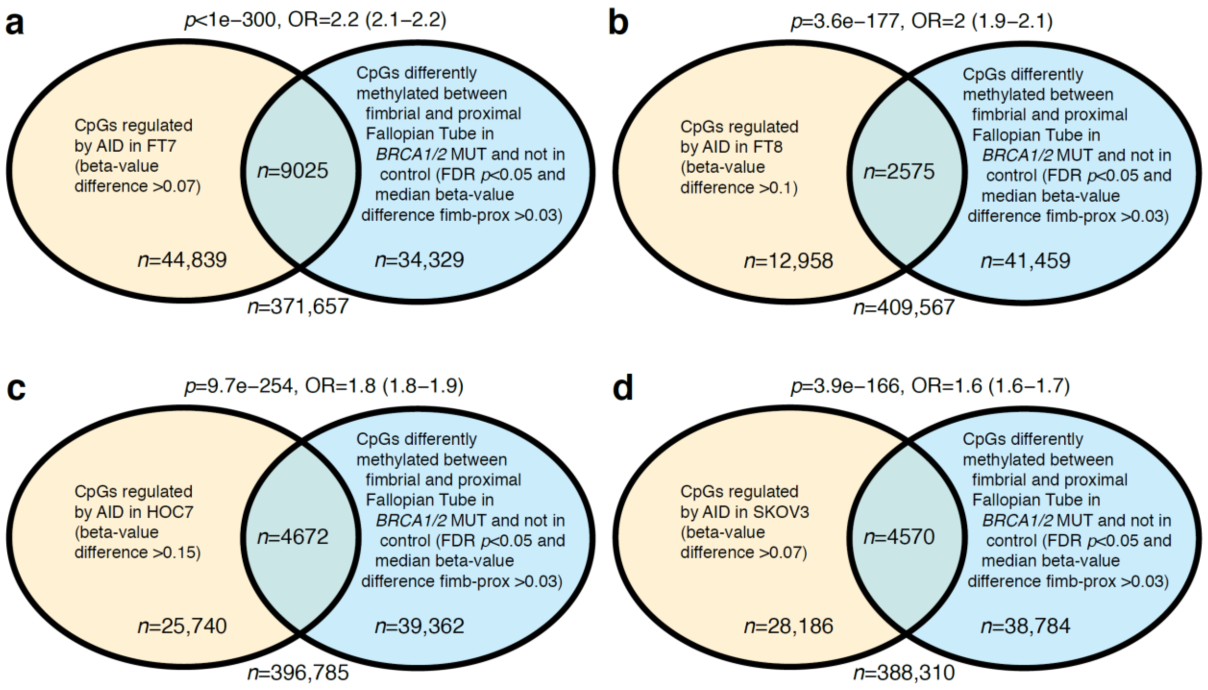

Supplementary Figure 10. Venn-diagrams illustrating significant overlap between CpGs that are differentially methylated between fimbrial and proximal Fallopian tube compartments in mutation carriers and cell-lines force-expressing AID (compared to empty vector control cells).

(a) FT7, delta-beta threshold = 0.07. (b) FT8, delta-beta threshold = 0.1. (c) HOC7, delta-beta threshold = 0.15. (d) SKOV3, delta-beta threshold = 0.07. The *p*-values were calculated using Fisher exact test.

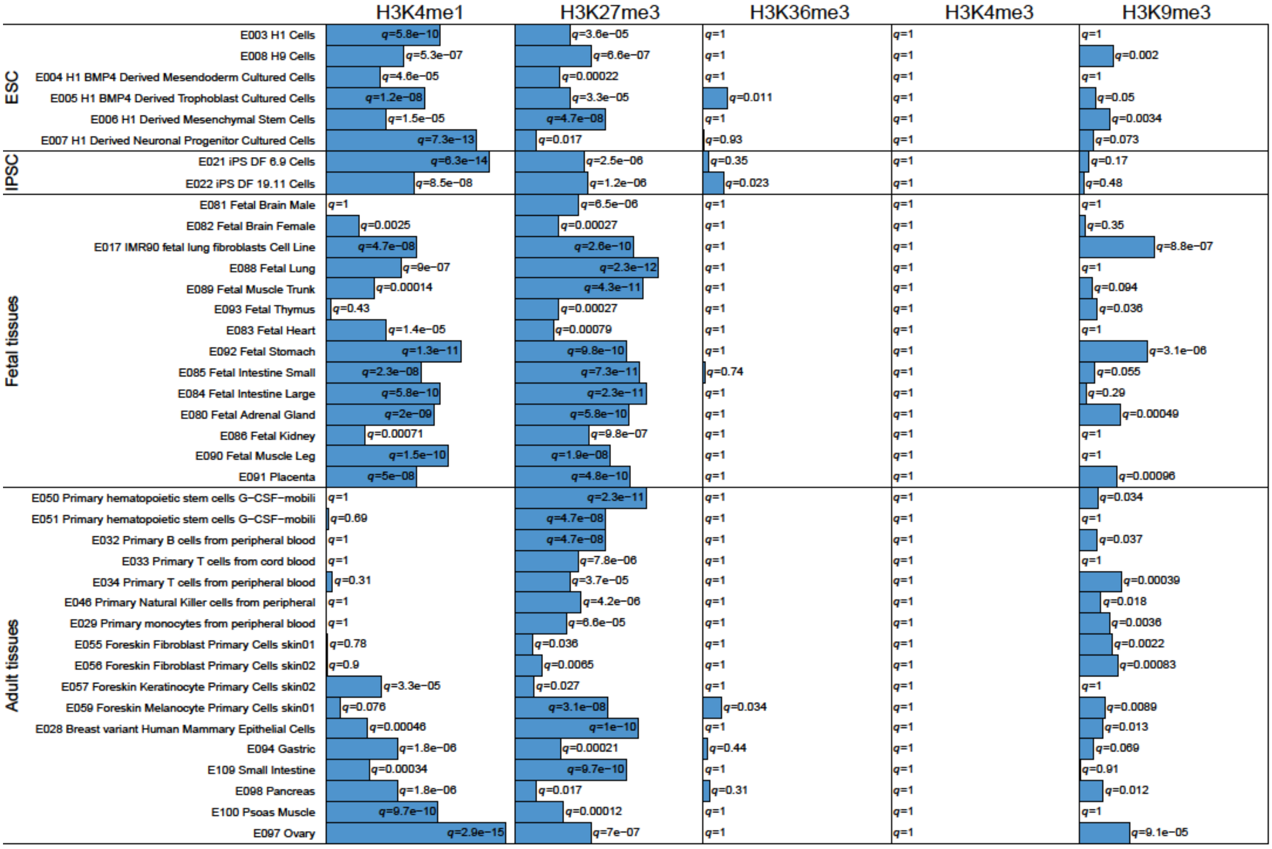

Supplementary Figure 11a

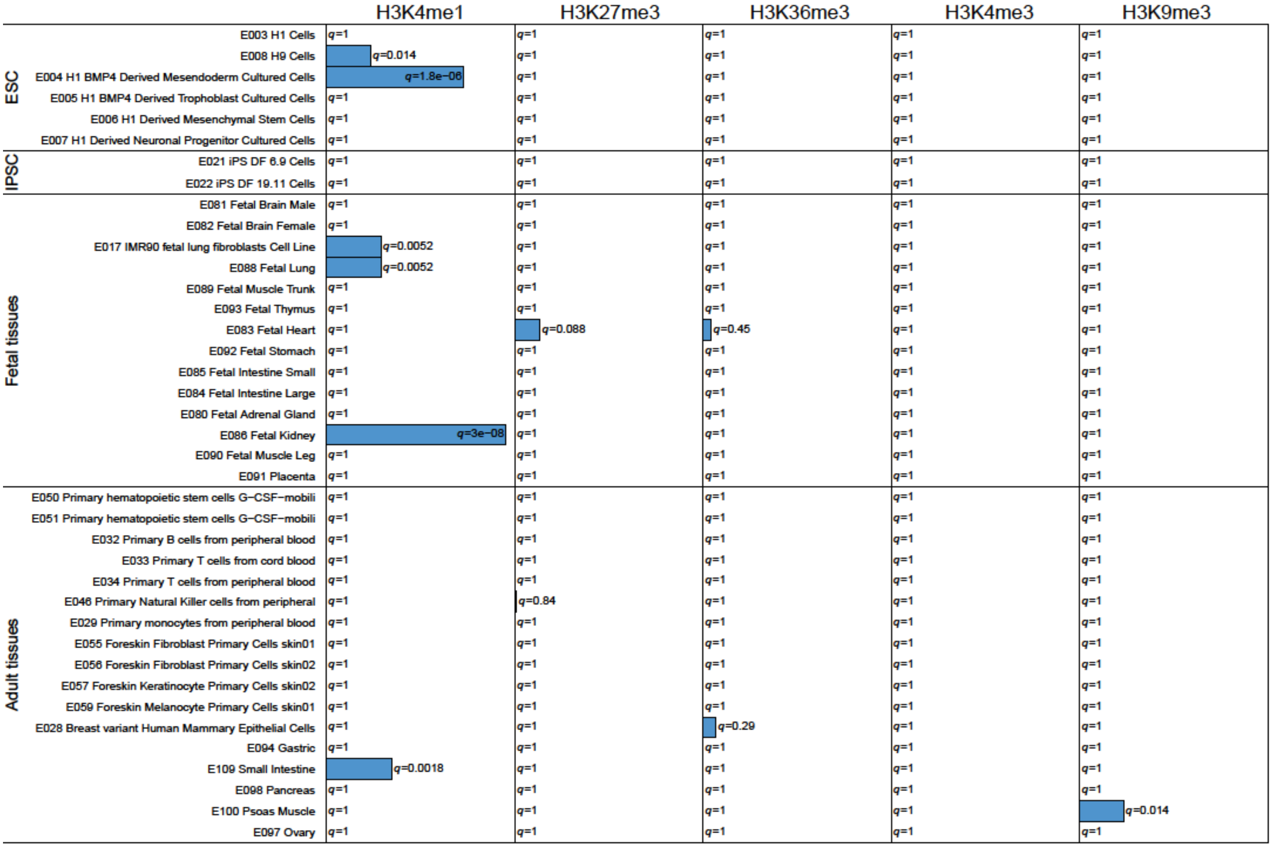

Supplementary Figure 11b

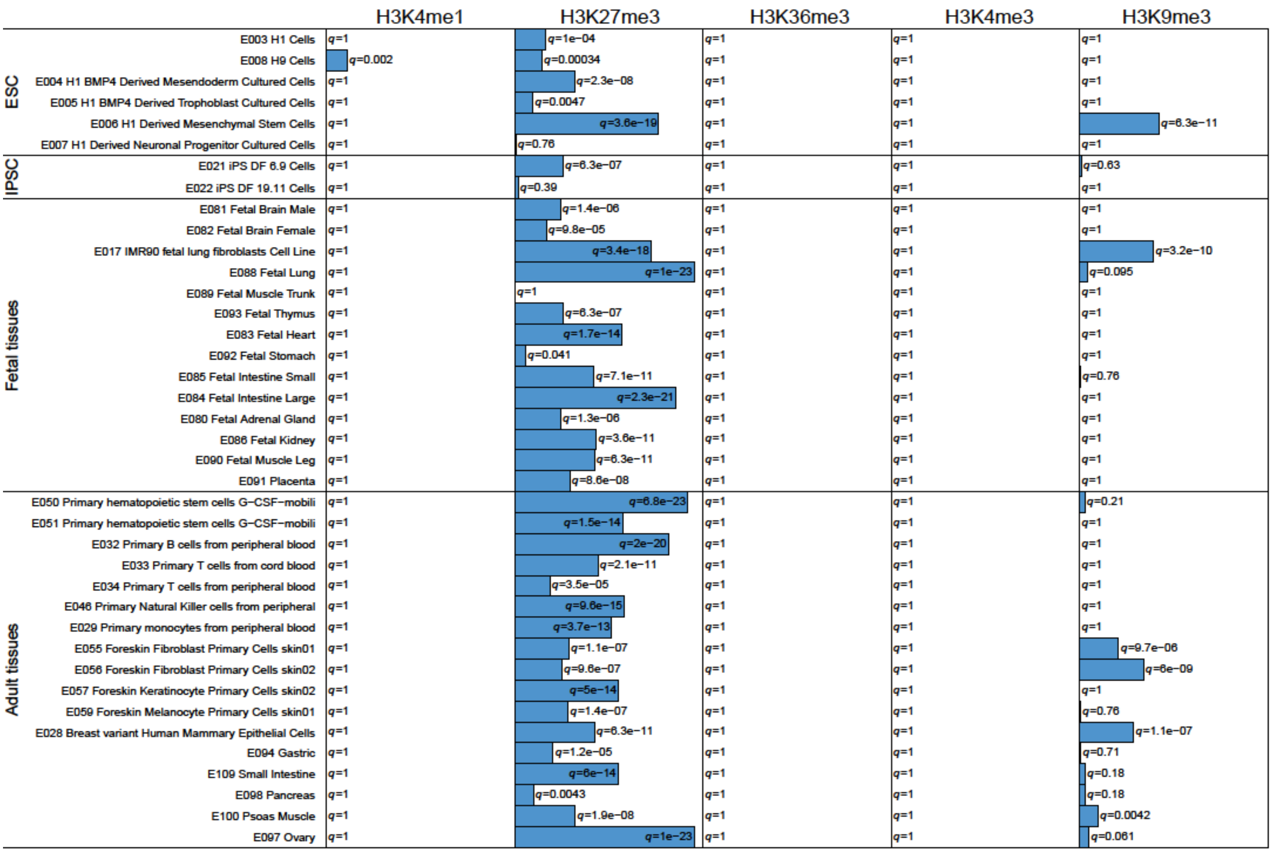

Supplementary Figure 11c

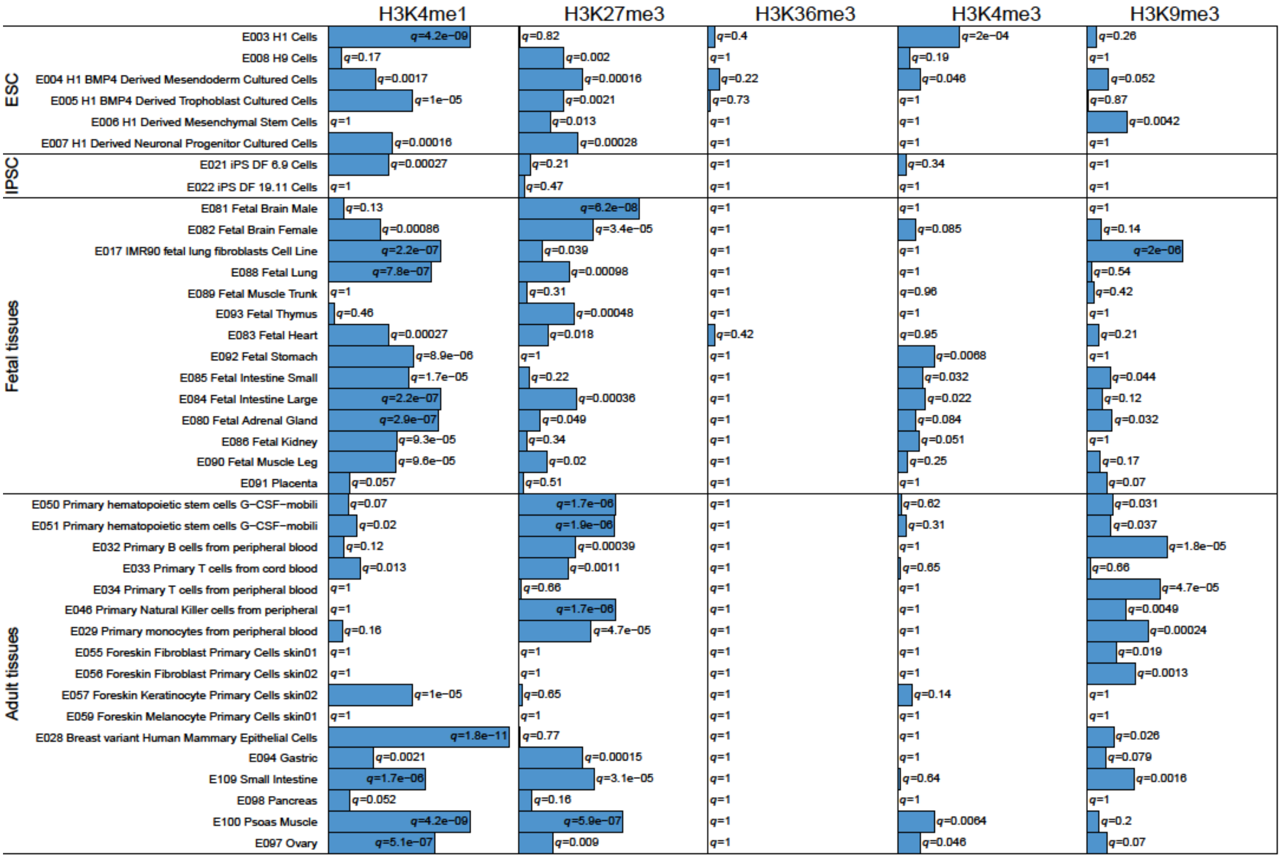

Supplementary Figure 11d

Supplementary Figure 11. Overlap with tissue specific patterns of histone modification

eFORGE *q*-values for enrichment of overlap for 1000 top CpGs hypomethylated upon AID overexpression in 4 cell types with H3K4me1, H3K27me3, H3K36me3, H3K4me3 and H3K9me3 peaks from Roadmap Epigenomics consolidated epigenomes. Cell types are (a) FT7, (b) FT8, (c) HOC7 and (d) SKOV3 cells.

For each set of test DMPs (differentially methylated positions), an overlap analysis is performed against the functional elements from either data source for each cell sample separately (ENCODE and Roadmap), and the number of overlaps is counted. A background distribution of the expected overlap counts for this DMP set is obtained by picking sets of the same number of DMPs as the test DMP set, matched for gene relationship and CpG island relationship annotation. The matched background sets are then overlapped with the functional elements and the background distribution of overlaps determined. By default 1000 matched sets are used. The enrichment value for the test DMP set is plotted as the -log10 (binomial *p*-value). The Benjamini-Yekutieli (BY) multiple-testing corrected *q*-value is evaluated to mark enrichments as significant at $q<0.05$.

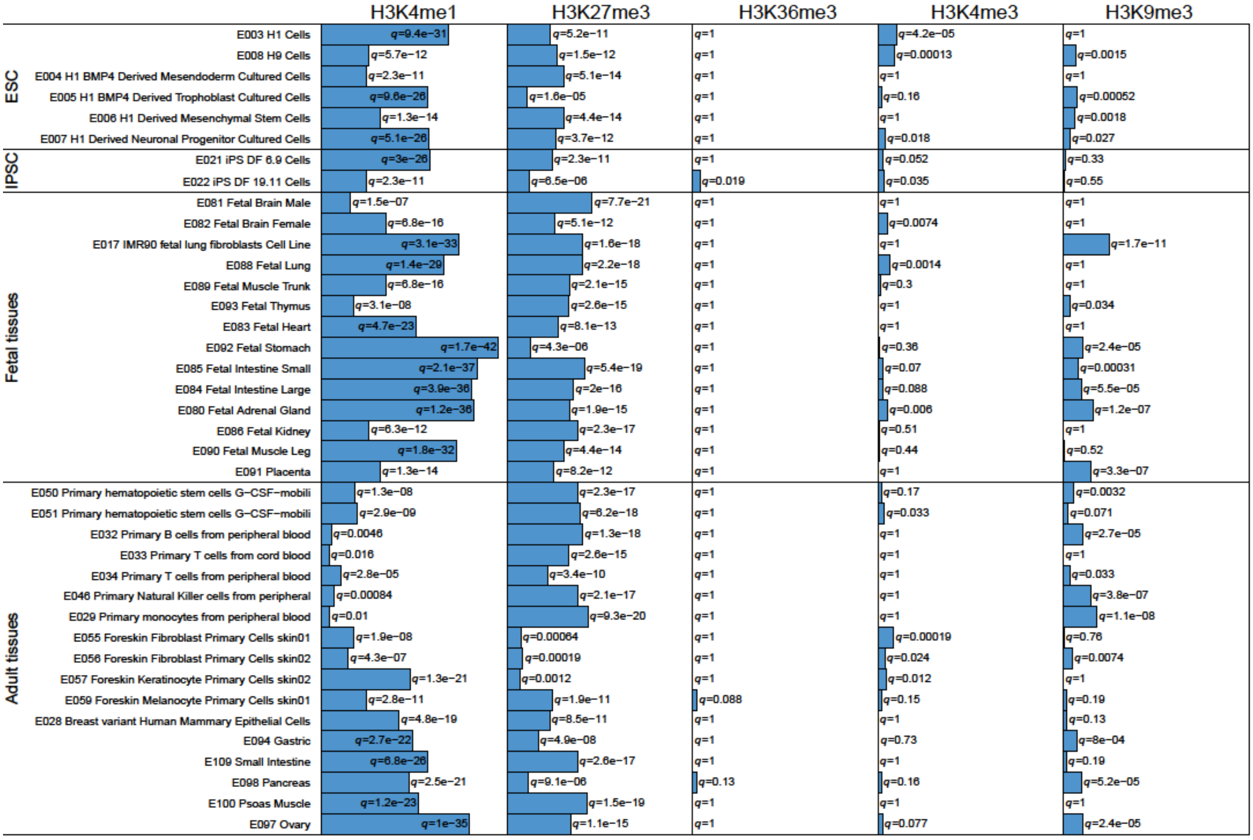

Supplementary Figure 12a

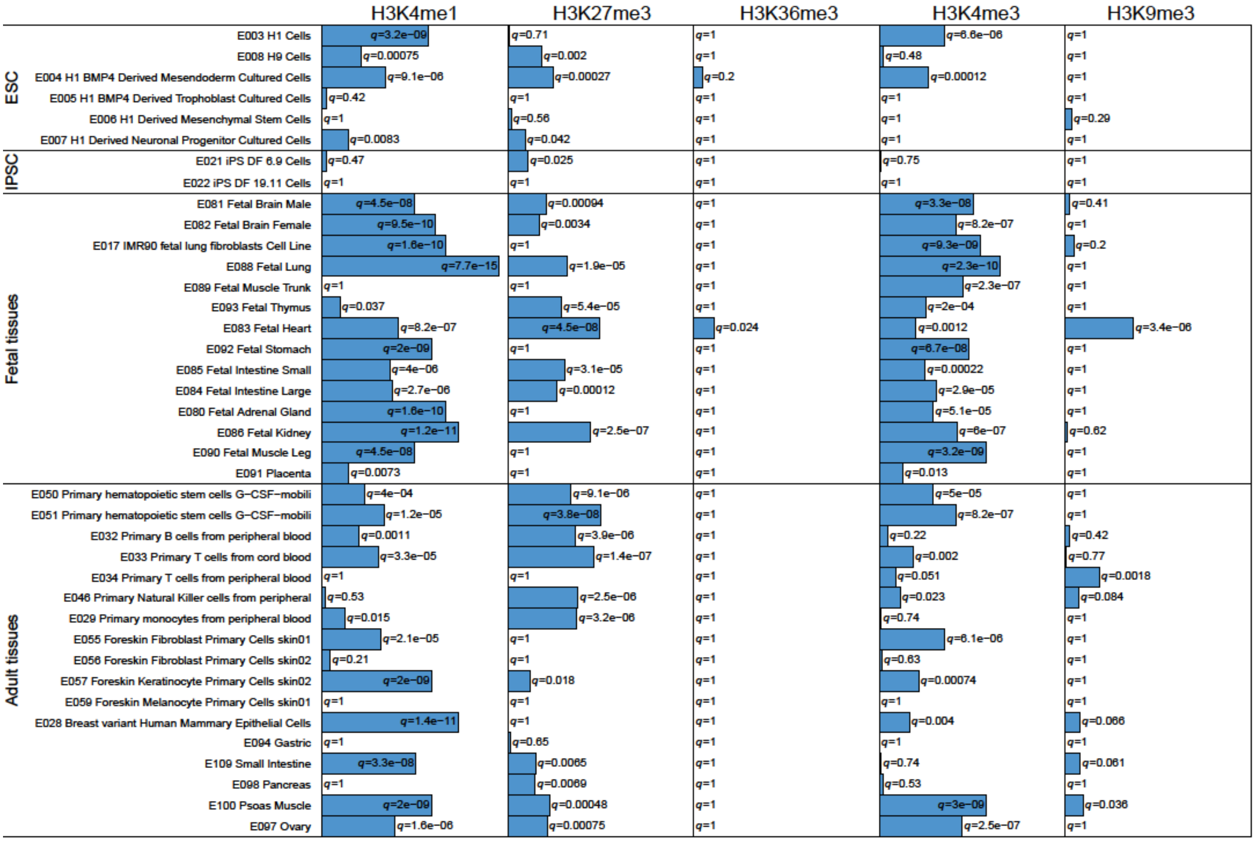

Supplementary Figure 12b

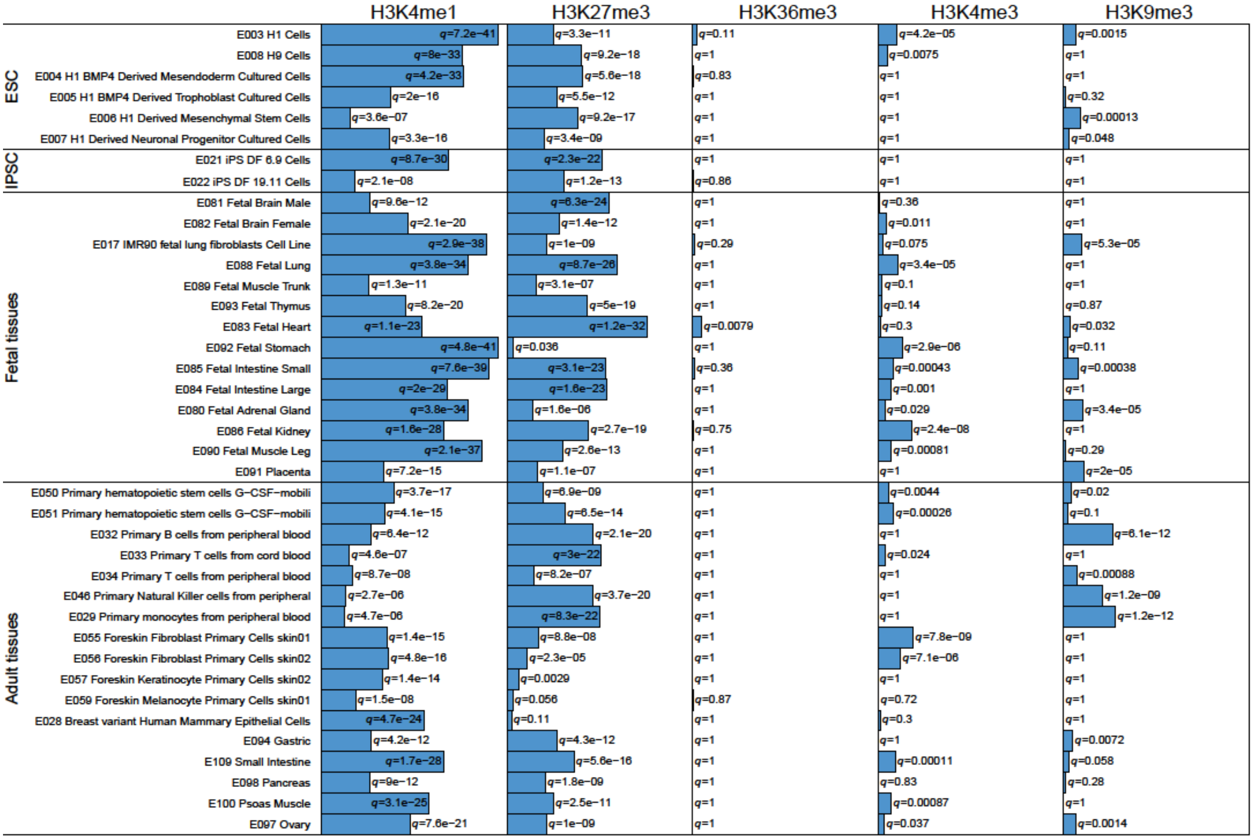

Supplementary Figure 12c

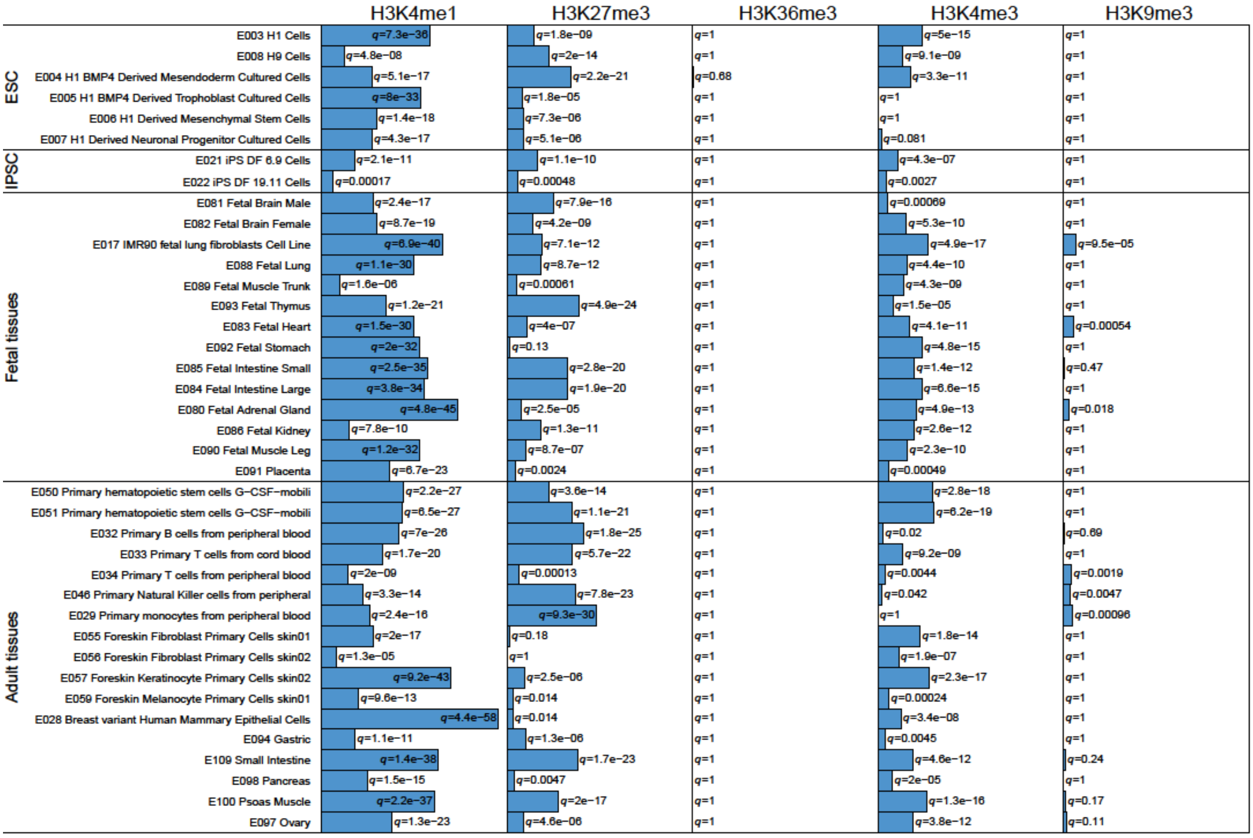

Supplementary Figure 12d

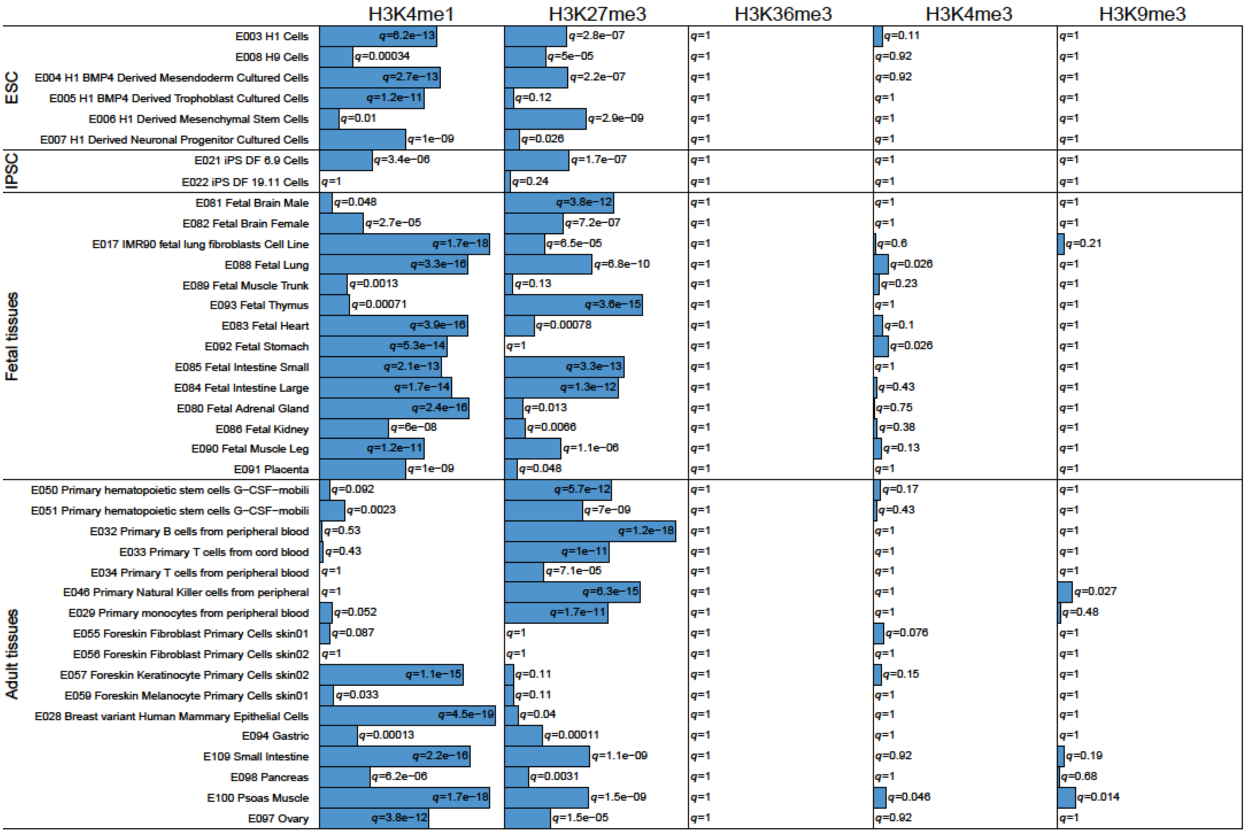

Supplementary Figure 12e

Supplementary Figure 12. Overlap with tissue specific patterns of histone modification

eFORGE *q*-values for enrichment of overlap for 1000 top CpGs hypermethylated upon AID overexpression in 4 cell types with H3K4me1, H3K27me3, H3K36me3, H3K4me3 and H3K9me3 peaks from Roadmap Epigenomics consolidated epigenomes. Cell types are (a) FT7, (b) FT8, (c) FT11, (d) HOC7 and (e) SKOV3 cells.

For each set of test DMPs (differentially methylated positions), an overlap analysis is performed against the functional elements from either data source for each cell sample separately (ENCODE and Roadmap), and the number of overlaps is counted. A background distribution of the expected overlap counts for this DMP set is obtained by picking sets of the same number of DMPs as the test DMP set, matched for gene relationship and CpG island relationship annotation. The matched background sets are then overlapped with the functional elements and the background distribution of overlaps determined. By default 1000 matched sets are used. The enrichment value for the test DMP set is plotted as the -log10 (binomial *p*-value). The Benjamini-Yekutieli (BY) multiple-testing corrected *q*-value is evaluated to mark enrichments as significant at $q<0.05$.

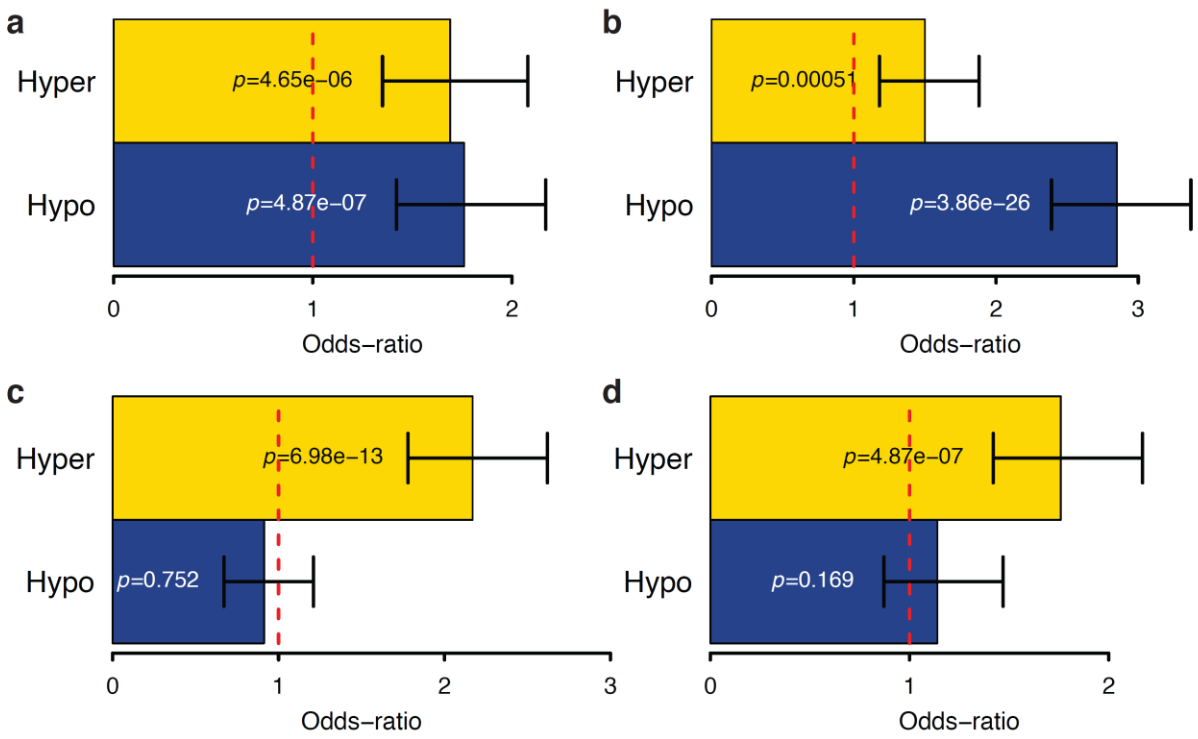

Supplementary Figure 13. Enrichment analyses

The 5000 CpGs with greatest beta increase and decrease, respectively, comparing non-serous (clear-cell, endometrioid and mucinous, *n*=36) to high-grade serous ovarian cancers (*n*=124) were tested for overlap with the 5000 CpGs showing the greatest beta increase and decrease, respectively, comparing (a) FT7, (b) FT8, (c) HOC7 and (d) SKOV3 cells force-expressing AID to those not force-expressing, equivalently to Figure 6c. *P*-values were obtained from Fisher's exact test; the error bars indicate 95% confidence intervals on the estimated odds-ratios.

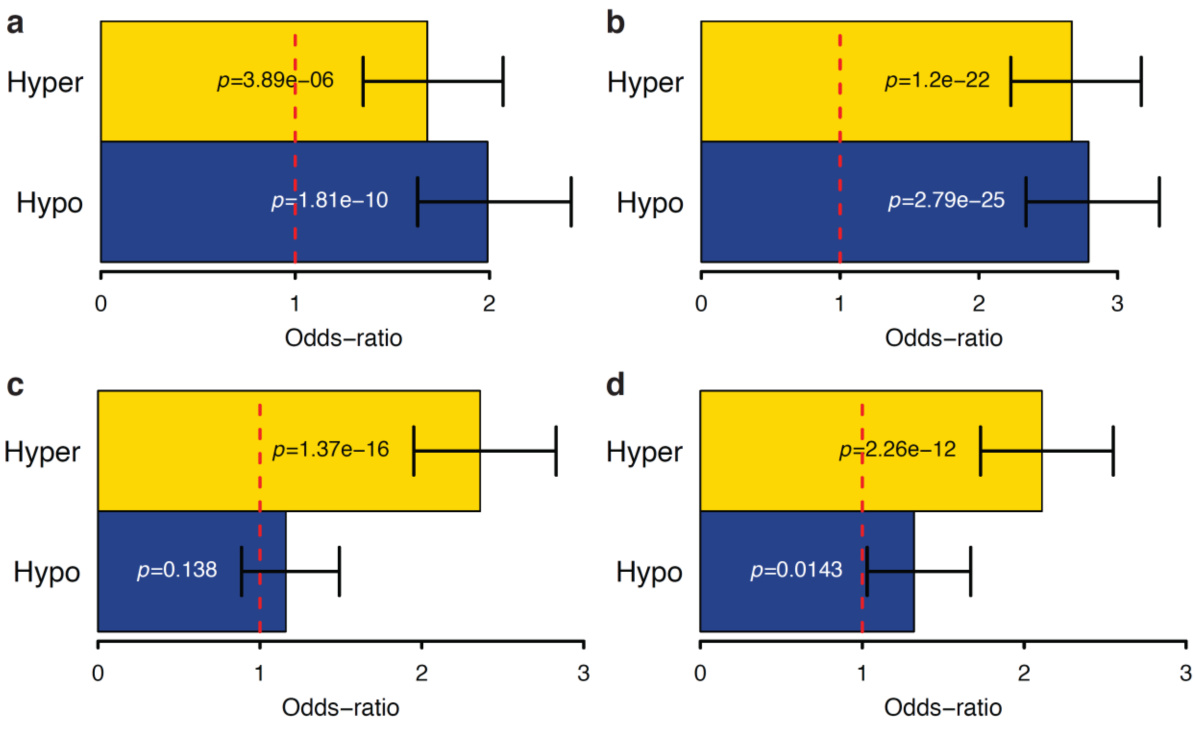

Supplementary Figure 14. Enrichment analyses

The 5000 most hyper- and 5000 most hypo-methylated fimbrial FT CpGs described in Figure 6a were tested for overlap between the 5000 CpGs with greatest beta increase and decrease, comparing (a) FT7, (b) FT8, (c) HOC7 and (d) SKOV3 cells force-expressing AID to those not force-expressing, equivalently to Figure 6d. *P*-values were calculated using Fisher's exact test; the error bars indicate 95% confidence intervals on the estimated odds-ratios.

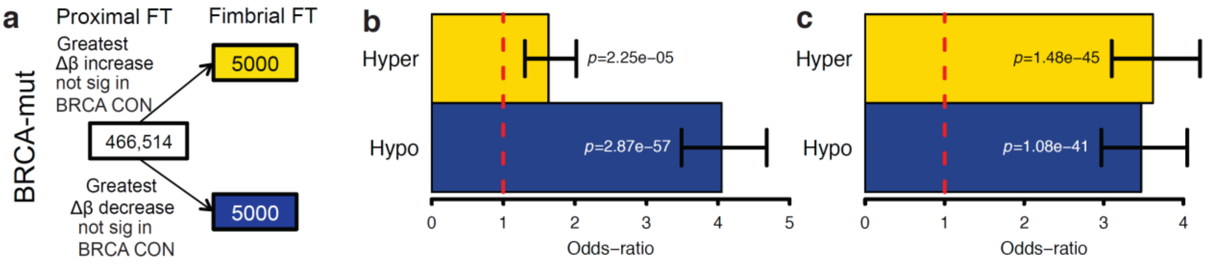

Supplementary Figure 15. Preservation of AID-mediated reprogramming in high grade serous cancers

(a) The 5000 CpGs with the most positive (i.e., hyper) and with the most negative (i.e., hypo) median delta-beta comparing fimb with prox in BRCA mutation carriers were selected, after first excluding all CpGs which are significant (*t*-test, FDR $p<0.05$) comparing fimb with prox in control women. (b) The overlap between the 5000 hyper- and hypomethylated CpGs described in (a) overlap highly significantly (*p*-values calculated with Fisher's exact test) with the top 5000 hyper- and hypomethylated CpGs (respectively) discriminating high grade serous from non-serous ovarian cancer types (as illustrated in Fig. 6a). (c) The top 5000 hyper- and hypomethylated CpGs described in (a) also overlap significantly (*p*-values calculated with Fisher's exact test) with the 5000 CpGs with greatest beta increase and decrease, respectively, comparing FT11 cell force-expressing AID to those not force-expressing (as illustrated in Fig 6a). All error bars indicate 95% confidence intervals on the estimated odds-ratios.

SUPPLEMENTARY TABLES:

**Gene/vector**	**Catalogue/Manufacturer**
pLenti-GIII-CMV-hAICDA	LV071677(Applied Biological Materials Inc.)
pLenti-GIII-CMV-hHOXC4	LV183502 (Applied Biological Materials Inc.)
pLenti-III-Blank vector	LV587 (Applied Biological Materials Inc.)
pBABE-puro-hTERT	addgene
HOXC4 MISSION shRNA	SHCLNG-NM_014620. TRCN0000436428 (Sigma-Aldrich)
HOXC4 MISSION shRNA	SHCLNG-NM_014620. TRCN0000435668 (Sigma-Aldrich)
HOXC4 MISSION shRNA	SHCLNG-NM_014620. TRCN0000018234 (Sigma-Aldrich)
HOXC4 MISSION shRNA	SHCLNG-NM_014620. TRCN0000018236 (Sigma-Aldrich)
HOXC4 MISSION shRNA	SHCLNG-NM_014620. TRCN0000018233 (Sigma-Aldrich)
MISSION® TRC2 pLKO.5-puro Empty Vector	SHC201 (Sigma-Aldrich)
Scrambled siRNA GFP Lentivector	LV015-G (Applied Biological Materials Inc.)
HOXC4-set siRNA/shRNA/RNAi Lentivector	i009944 (Applied Biological Materials Inc.)
HOXC4, Silencer ® Select Pre-designed siRNA	siRNA ID; s6816 (Thermo Fisher Scientific)

Supplementary Table 1: Details of constructs used for gene overexpression or knock down experiments

**Gene**	**Primer sequence**
HOXC4 forward	GCCAGCAAGCAACCCATAGT
HOXC4 reverse	CCTTCTCCTTCGGGTCAGGT
AID forward	CCGGGAAACTGTGGCGTGATGG
AID reverse	AGGTGGAGGAGTGGGTGTCGCTGTT
GAPDH forward	CCGGGAAACTGTGGCGTGATGG
GAPDH reverse	AGGTGGAGGAGTGGGTGTCGCTGTT
PAX8 forward	ATCCGGCCTGGAGTGATAGG
PAX8 reverse	TGGCGTTTGTAGTCCCCAATC
CK7 forward and reverse	Inventoried primers from life technologies

Supplementary Table 2: Details of primers used for real time PCR
